# Decoding the Cellular Heterogeneity and Malignant Progression of Human Penile Squamous Cell Carcinoma by Single‐Cell RNA Sequencing

**DOI:** 10.1002/advs.202503894

**Published:** 2026-02-04

**Authors:** Xiheng Hu, Wensheng Shi, Liang Dong, Lingjuan Huang, Xiyuan Zhang, Yiting Feng, Jie Sun, Lanlan Liu, Teng Liu, Jun Fu, Bowen Zhong, Qihao Leng, Xiaohua Wu, Minfeng Chen, Lingfang Li, Yuan Li, Xin Jin, Long Wang, Jian Cao, Xin Li, Mingzhu Yin, Xiang Chen

**Affiliations:** ^1^ Department of Urology Xiangya Hospital Central South University Changsha Hunan China; ^2^ National Engineering Research Center of Personalized Diagnostic and Therapeutic Technology Central South University Changsha Hunan China; ^3^ Furong Laboratory Changsha Hunan China; ^4^ Department of Dermatology Hunan Engineering Research Center of Skin Health and Disease Hunan Key Laboratory of Skin Cancer and Psoriasis Xiangya Hospital Central South University Changsha Hunan China; ^5^ Department of Urology the Second Xiangya Hospital Central South University Changsha China; ^6^ Clinical Research Center (CRC) Medical Pathology Center (MPC) Cancer Early Detection and Treatment Center (CEDTC) and Translational Medicine Research Center (TMRC) Chongqing University Three Gorges Hospital Chongqing University Chongqing China; ^7^ School of Medicine Chongqing University Chongqing China; ^8^ CQU ‐ Ferenc Krausz Nobel Laureate Scientific Workstation Chongqing University Three Gorges Hospital & Academy for Advanced Interdisciplinary Technology Chongqing China; ^9^ Department of Cardiovascular Medicine Xiangya Hospital Central South University Changsha China; ^10^ Department of Urology The Third Xiangya Hospital of Central South University Changsha Hunan China; ^11^ Department of Urology The Affiliated Cancer Hospital of Xiangya School of Medicine Central South University/Hunan Cancer Hospital Changsha China

**Keywords:** PSCC, SEMA3C, SEMA3C^high^ Mals, single‐cell RNA sequencing, TME

## Abstract

Penile squamous cell carcinoma (PSCC) is a rare genitourinary malignancy, and factors of its tumor microenvironment (TME) could serve as prognostic indicators for tumor recurrence and metastasis. Here, we generated a comprehensive single‐cell map of PSCC (66 421 cells) and identified 9 distinct cell populations with samples from nine tumor samples and six adjacent normal samples. Among the malignant cells, SEMA3C^high^ Mals was found to be associated with epithelial‐mesenchymal transition. T cells in tumor tissues are in a highly exhausted state, while SPP1^high^ TAMs were observed to promote tumor progression. Cancer‐associated fibroblasts were found to interact with malignant cells to facilitate EMT through several pathways. Notably, there is a specific type of pericyte called POSTN^+^ pericytes, which can promote angiogenesis and extracellular matrix remodeling in PSCC. Finally, SEMA3C was identified as an effective biomarker reflecting cancer stage and microvessel density. Overall, we investigated the heterogeneity of TME from a single‐cell perspective and demonstrated that SEMA3C serve as an effective biomarker for predicting lymph node metastasis and prognosis in PSCC. These findings may offer valuable insights for future therapeutic strategies.

## Graphical abstract

Single‐cell transcriptomic and functional analyses identify SEMA3C as a key regulator of tumor progression and tumor microenvironment remodeling in penile squamous cell carcinoma. SEMA3C promotes epithelial–mesenchymal transition, tumor growth, and invasion while shaping an immunosuppressive microenvironment, highlighting its potential as a prognostic biomarker and therapeutic target.

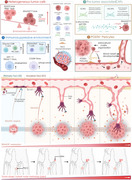

## 1 Introduction

Penile squamous cell carcinoma (PSCC) is a rare cancer with a prevalence of 0.1‐1 case per 100 000 men in developed countries, and it accounts for up to 10% of malignancies in some low‐income regions [1]. Several risk factors for PSCC have been identified, including poor hygiene, circumcision, phimosis, HIV infection, smoking and low socioeconomic status [1]. To date, limited strategies (such as surgery, radiation and chemotherapy) have been applied in PSCC treatment, and they are usually noncurative for patients with metastatic or advanced carcinoma [2, 3, 4, 5]. Surprisingly, a diverse array of immune cells and immune‐associated markers in PSCC have been demonstrated to serve as effective indicators for predicting recurrence and survival [6], implying that immune‐based therapy is a promising strategy. However, current knowledge regarding the tumor microenvironment (TME) and critical pathogenic pathways is limited in PSCC. Therefore, understanding the intricate nature of the TME in PSCC is crucial for early diagnosis and the development of therapeutic targets.

The TME includes diverse tumor cells, immune cells and stromal cells, which play a key role in the initiation and progression of tumors [7, 8, 9]. Immune cells and stromal cells might interact with tumor cells to influence the effect of antitumor therapy [10, 11]. Single‐cell RNA sequencing (scRNA‐seq) provides unprecedented resolution for TME exploration [12], increasing the recognition of PSCC heterogeneity. Recent studies employing single‐cell and spatial transcriptomics have begun to reveal the complex cellular landscape of PSCC. For example, single‐cell transcriptomic profiling has revealed that TP53 mutations may drive aggressive tumor phenotypes independent of HPV status [13], while spatial and single‐cell analyses have highlighted dynamic shifts in tumor differentiation and immune infiltration [14]. Despite these emerging insights, the biological mechanisms driving PSCC progression, particularly those involving TME interactions, are still being elucidated. The complex cellular composition of PSCC likely reflects underlying functional heterogeneity among tumor cells, including variations in stemness, invasiveness, and immune modulation. These functional differences are shaped by specific molecular regulators that may drive tumor progression and offer opportunities for therapeutic intervention [15, 16]. Elucidating these regulators could yield mechanistic insights and inform biomarker discovery [17].

Herein, we applied scRNA‐seq to characterize the TME of PSCC at single‐cell resolution. Our study aimed to dissect the cellular heterogeneity of malignant, immune, and stromal compartments, to reveal potential regulators of tumor progression and immune suppression. In particular, we focused on identifying cell states and signaling pathways, such as those involving cancer‐associated fibroblasts, tumor‐associated macrophages, and immune checkpoints, that may serve as biomarkers or therapeutic targets.

## 2 Results

### 2.1 Elucidating the Cellular Ecosystem of Human PSCC

**FIGURE 1:**
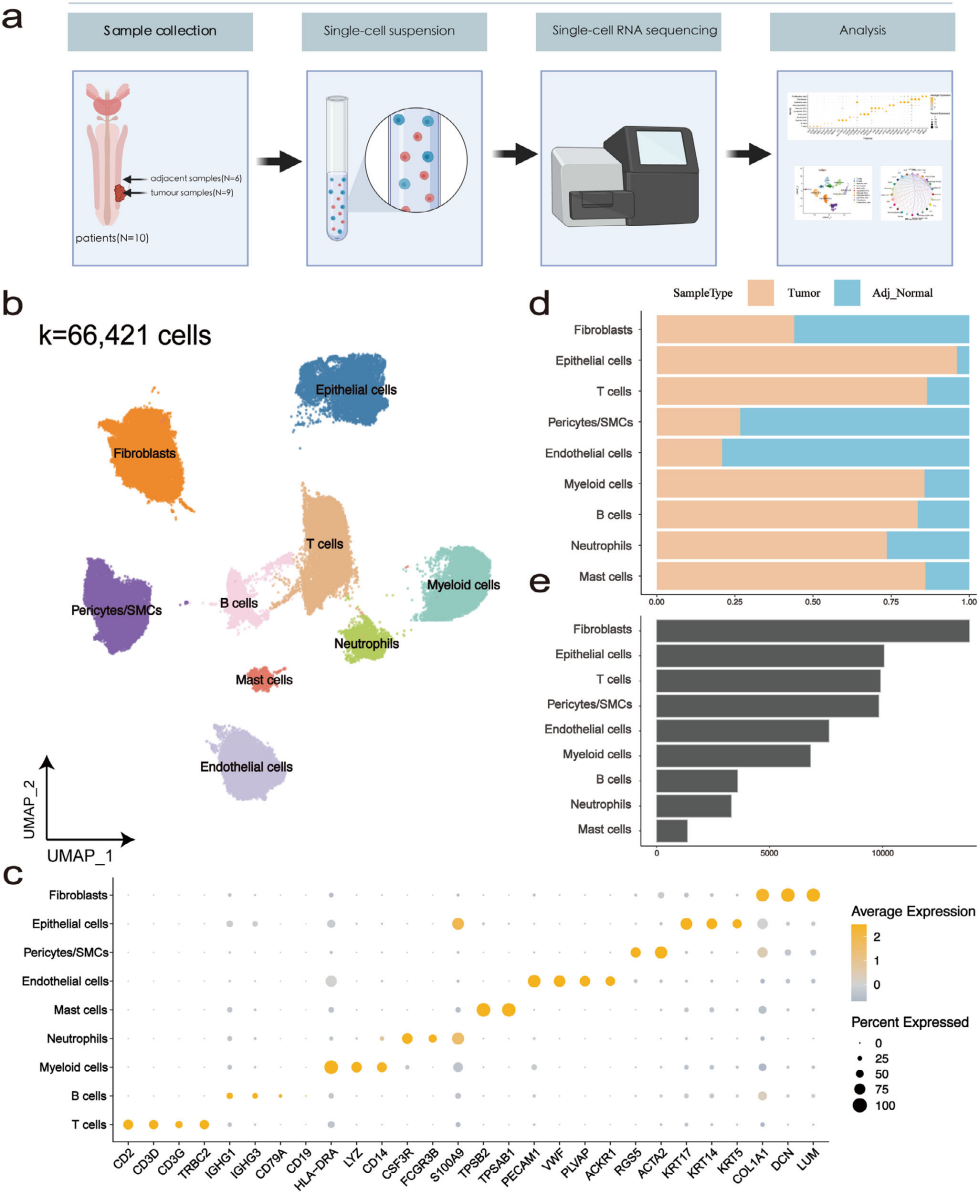
Construction of the PSCC atlas at the single‐cell resolution. (a) Schematics of overall study design. (b) UMAP embedding of 66 421 high‐quality cells, colored by cell type. (c) Dot plot showing log‐normalized expression values derived from Seurat's NormalizeData() function. Colors indicate mean expression (log1p‐transformed), and dot size reflects the proportion of expressing cells. (d) The proportion of cells from each sample type in each cluster. (e) The number of cells in each cluster.

To elucidate the cellular atlas of PSCC, nine tumor samples and six adjacent normal samples were surgically obtained from ten patients and immediately processed for scRNA‐seq using the BD, Singleron and Seekone platform (Figure 1a; Table S1). After stringent quality control, we obtained single‐cell transcriptomes from a total of 66 421 cells and retained 53 224 genes for downstream analyses (Figure 1b). The gene expression level was regressed against read depth and mitochondrial read count, and we next corrected for batch effects using the Harmony algorithm (Figure S1a,b) [18]. Afterward, we employed the Harmony‐corrected principal components to generate a unified uniform manifold approximation and projection (UMAP) embedding space and then performed graph‐based clustering and annotated each cluster using their canonical cell markers. Finally, nine main cell types were identified, including fibroblasts, epithelial cells, T cells, pericytes/smooth muscle cells (SMCs), endothelial cells, myeloid cells, B cells, neutrophils, and mast cells (Figure 1 b,c). We discovered that the cell type proportion was highly variable among diverse sample types (Figure 1d; Figure S1c), indicating a significant difference in the TME between the tumor and the adjacent tissue. Subsequently, we conducted a more in‐depth analysis among epithelial cells, T cells, myeloid cells, fibroblasts, endothelial cells and pericytes/SMCs, which are major components of the microenvironment in PSCC (Figure 1e).

### 2.2 Heterogeneity and Characteristics of Epithelial Subpopulations in the PSCC Microenvironment

**FIGURE 2:**
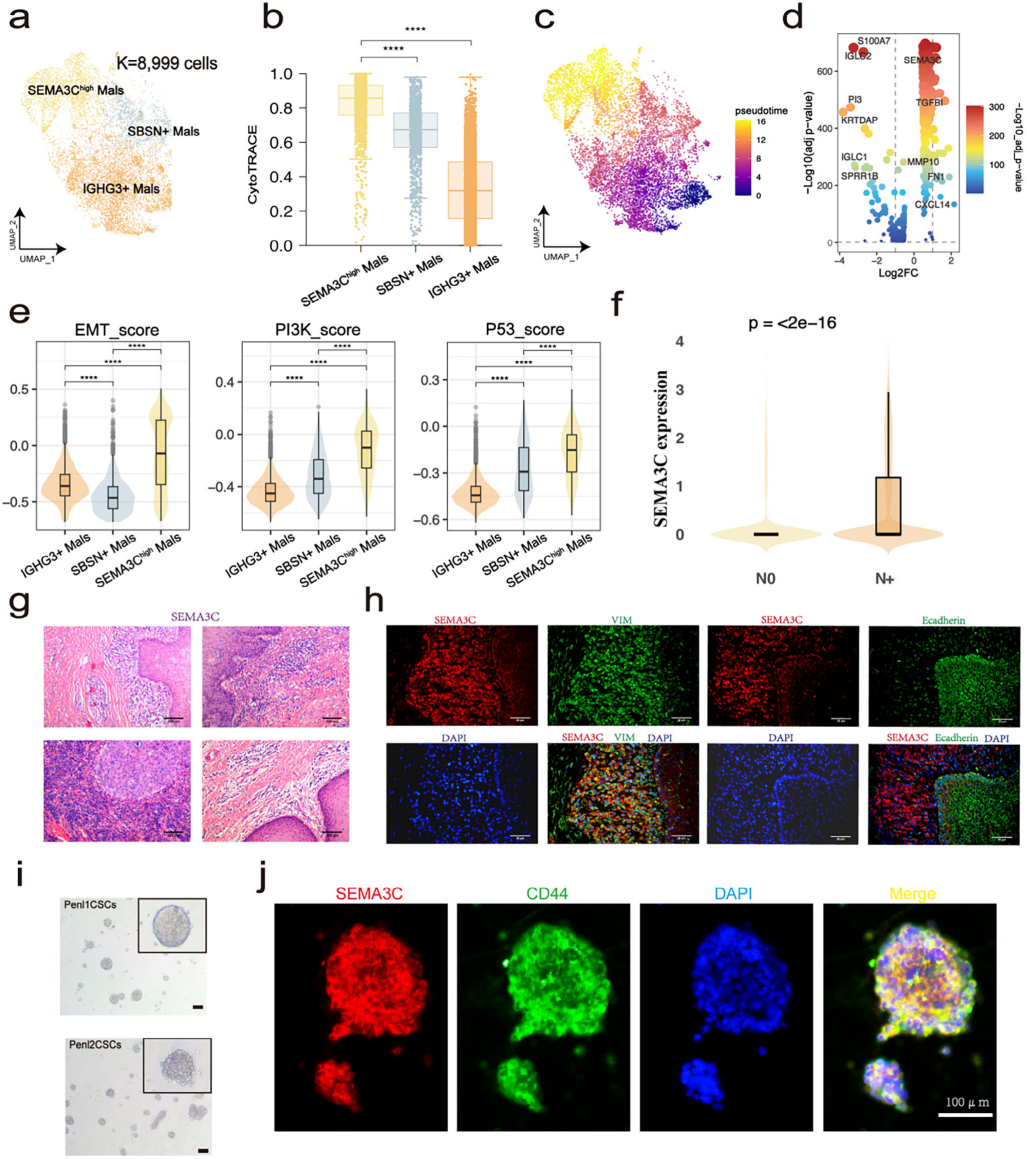
Identifying and analyzing malignant cell in epithelial cell. (a) UMAP of three malignant cells. (b) Distribution of CytoTRACE score of each cell type, ranking by the median value. (c) Trajectory of differentiation from IGHG3^+^ Mals into SEMA3C^high^ Mals by Monocle3 analysis. (d) Volcano plot showing the differential expressed genes between SEMA3C^high^ Mals and other Mals. (e) Comparison of EMT score, PI3K score and P53 score among three kinds of Mals, Wilcoxon signed‐rank test, ^****^ *p* < 0.0001. (f) Comparison of SEMA3C expression levels between node‐negative (N0) and node‐positive (N+) PSCC patients, expression values were computed using Seurat's NormalizeData() function with log1p transformation, Wilcoxon signed‐rank test, ^****^ *p* < 0.0001. (g) The H&E staining in representative samples. (h) Immunofluorescence co‐staining of SEMA3C with mesenchymal and epithelial markers in representative PSCC samples. Left panel: Co‐staining of SEMA3C (red) with Vimentin (VIM) (green), a mesenchymal marker. Right panel: Co‐staining of SEMA3C (red) with E‐cadherin (green), an epithelial marker. Nuclei were counterstained with DAPI (blue). (i) Human penile cancer cell lines were utilized to cultivate penile cancer tumor spheres. (j) The immunofluorescence co‐staining of SEMA3C and CD44 in penile cancer tumor spheres (SEMA3C: red; CD44: green; DAPI: blue).

To explore the variation in expression states among epithelial cells, the 8 999 epithelial cells were further sub‐clustered and identified as malignant based on inferCNV results and the expression of known representative malignancy‐associated genes (Figure 2a; Figure S2a,b, Materials and Methods). CytoTRACE results revealed that SEMA3C^high^ Mals had the highest stemness score among the three subgroups (Figure 2b, Materials and Methods). In addition, Monocle3 analysis indicated that IGHG3^+^ Mals were prone to differentiate into SEMA3C^high^ Mals (Figure 2c, Materials and Methods). To assess the consistency between Monocle3 and CytoTRACE, we performed a correlation analysis between Monocle3‐inferred pseudotime and CytoTRACE differentiation scores (Figure S2c). A strong positive correlation was observed (Pearson R = 0.73, *p* < 0.001), suggesting that both methods captured a similar trajectory of cellular progression. Furthermore, SEMA3C^high^ Mals tended to score higher in most of the “hallmarks of cancer” terms, such as “Self‐sufficiency in growth signals” and “Insensitivity to antigrowth signals”, further supporting that SEMA3C^high^ Mals pocess the highest levels of malignancy compared to the other two clusters (Figure S2d, Materials and Methods). To gain insights into the heterogeneity of malignant cells, we did differential expression analysis and results suggested that EMT‐related genes, such as TGFBI, FN1, SEMA3C, and MMP‐related genes, were upregulated in SEMA3C^high^ Mals (Figure 2d). Next, we assessed the EMT activity, PI3K pathway activity, and p53 pathway activity scores for each cell group (Materials and Methods). These pathways, which are critically involved in PSCC progression and aggressiveness, showed consistently elevated activity in SEMA3C^high^ Mals, indicating their potential contribution to the aggressive phenotype of this subpopulation (Figure 2e) [13, 19, 20]. Given the potential impact of clinical heterogeneity, especially nodal involvement (N stage), on the TME, we further stratified our cohort into node‐negative (N0) and node‐positive (N+) groups. Consistently, we observed that SEMA3C expression was significantly elevated in the N+ cases (Figure 2f), with SEMA3C^high^ Mals more enriched in node‐positive patients (Figure S2e). Moreover, functional scores such as EMT and PI3K pathway activities were also higher in the N+ samples, whereas p53 signaling showed no significant difference (Figure S2f). These findings further highlight that nodal status contributes to the heterogeneity of TME in PSCC. We subsequently performed H&E staining to validate the expression of SEMA3C in a clinical cohort, and observed the presence of SEMA3C‐positive cells within the tumor tissue (Figure 2g). We defined the area containing the primary foci as IS (in situ) and the area with invasive foci as IV (invasive). In immunofluorescence costaining experiments, IV region exhibited increased expression level of SEMA3C and VIM, and reduced expression level of some epithelial signature‐related proteins (such as Ecadherin), implying that tumor cells with high expression of SEMA3C are predominantly located within invasive foci (Figure 2h). To further investigated the regulatory role of SEMA3C in penile cancer in vitro, human penile cancer cell lines, Penl1 and Penl2, were utilized to cultivate penile cancer tumor spheres (Figure 2i, Materials and Methods). Afterward, we observed that SEMA3C, VIM, CD44, and FN1 were expressed in the tumor spheres, while E‐Cadherin was weakly expressed (Figure S2g). Previous studies have identified CD44^+^ penile cancer tumor cells as tumor stem cells [21]. Immunofluorescence staining of SEMA3C and CD44 suggested that SEMA3C may also serve as a marker gene for penile cancer stem cells (Figure 2j).

### 2.3 T Cells and Myeloid Cells Together Form the Immunosuppressive Microenvironment of PSCC

**FIGURE 3:**
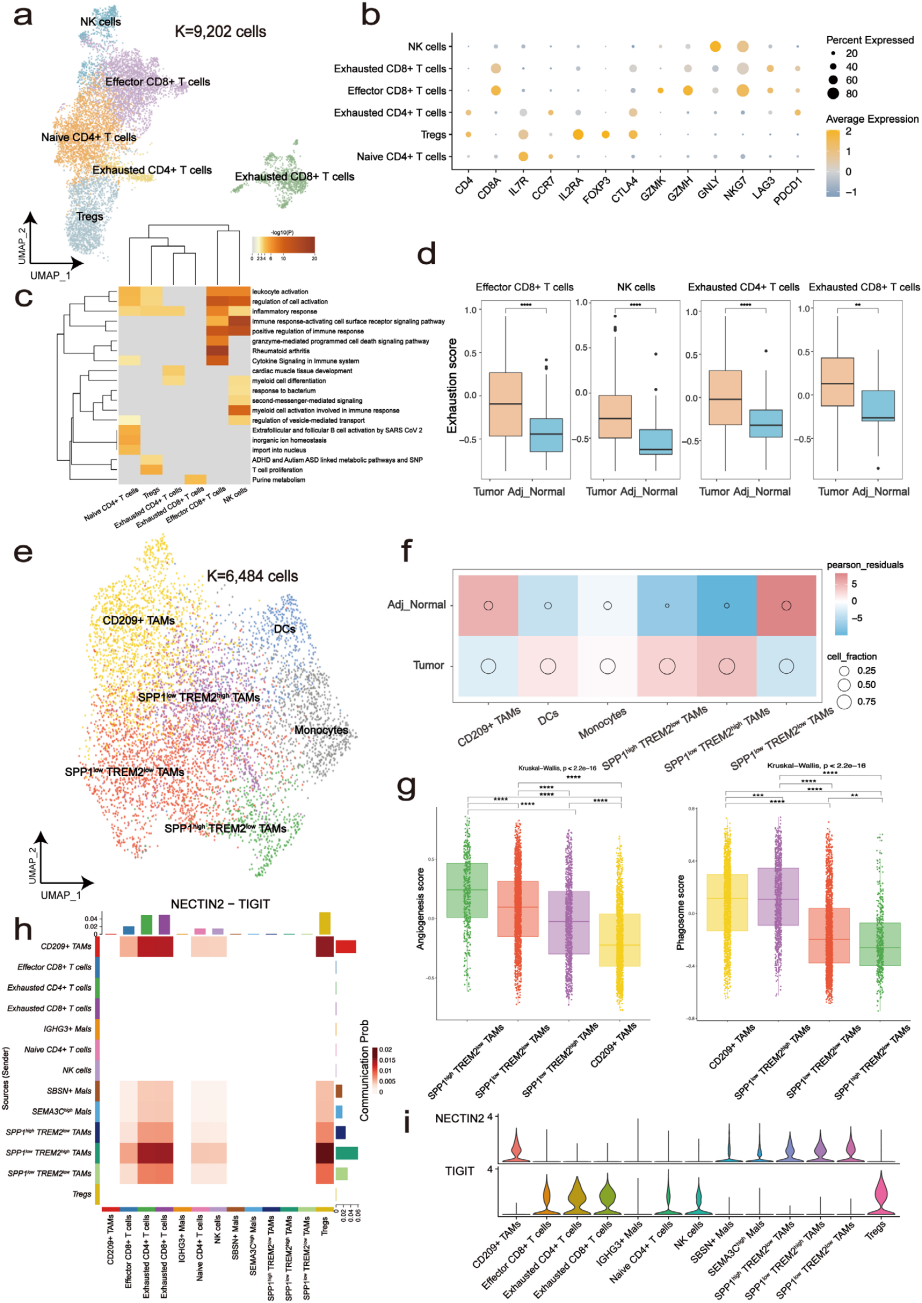
Overview of immune microenvironment in PSCC. (a) UMAP of different subsets in T cells. (b) Dot plot showing log‐normalized expression values derived from Seurat's NormalizeData() function. Colors indicate mean expression (log1p‐transformed), and dot size reflects the proportion of expressing cells. (c) Functional enrichment analysis of six kinds of T cells. (d) Exhaustion scores of effector CD8^+^ T cells, NK cells, and exhausted T cells across different sample types. Scores were calculated using the GSVA R package based on a curated exhaustion‐related gene set and are presented as unitless enrichment values. Statistical significance was assessed using the Wilcoxon signed‐rank test. ^*^ *p* < 0.05, ^**^ *p* < 0.001, ^***^ *p* < 0.0001. (e) UMAP of different subsets in myeloid cells. (f) Pearson residuals and cell fraction of each cell cluster in different sample types. (g) Comparison of angiogenesis and phagocytosis score among four kinds of TAMs, ranking by the median value. (h) NECTIN2‐TIGIT pathway communication probability between different cell types. (i) Expression of the NECTIN2‐TIGIT ligand‐receptor pair across different cell types. Expression values were computed using Seurat's NormalizeData() function with log1p transformation.

The immune cells within the TME can act as a double‐edged sword and play an important role in tumorigenesis [22]. We subsequently dissected the function of T cells and myeloid cells in PSCC. Overall, six T cell subpopulations were detected: naive CD4^+^ T cells (IL7R^+^, FOXP3^−^), regulatory T cells (Tregs, IL2RA^+^, FOXP3^+^, CTLA4^+^, ICOS^+^), exhausted CD4^+^ T cells (CD4^+^, CTLA4^+^, PDCD1^+^), effector CD8^+^ T cells (CD8A^+^, GZMK^+^), exhausted CD8^+^ T cells (CD8A^+^, GZMH^−^, LAG3^+^, PDCD1^+^), natural killer cells (NK cells, CD8^−^, GNLY^+^, NKG7^+^) (Figure 3a,b). Functional enrichment analysis reveals that the immune effector functions of exhausted CD4^+^ and CD8^+^ T cells are significantly impaired (Figure 3c). The distribution of T cell varied significantly across different tissues. Exhausted CD8^+^ T cells, effector CD8^+^ T cells, and Tregs were enriched in tumor tissue. In contrast, naive CD4^+^ T cells had a low infiltration (Figure S3a). This differential distribution may enable the tumor to evade detection and destruction by the immune system, thereby promoting tumor growth and progression. Additionally, we observed that exhausted CD4^+^ T cells, effector CD8^+^ T cells, exhausted CD8^+^ T cells, and NK cells all displayed a consistent upregulation of exhaustion scores in PSCC (Figure 3d). It is noteworthy that the expression of exhaustion‐related genes varies significantly across different cell types. In CD8^+^ T cells, LAG3, PDCD1, and HAVCR2 are expressed at higher levels in tumor samples compared to adjacent non‐cancerous tissues. Additionally, in exhausted T cells, the expression of LAG3, MYO7A, and HAVCR2 is even more elevated in tumor samples (Figure S3b).

Furthermore, subclustering of myeloid cells identified six subtypes: SPP1^high^ TREM2^low^ TAMs (SPP1^high^, TREM2^low^, CD163^+^, MRC1^+^, CCL2^−^), SPP1^low^ TREM2^high^ TAMs (SPP1^low^, TREM2^high^, CD163^+^, MRC1^+^), SPP1^low^ TREM2^low^ TAMs (SPP1^low^, TREM2^low^, CD163^+^, MRC1^+^), CD209^+^ TAMs (CD209^+^, CD163^+^, MRC1^+^), monocytes (FCN1^+^, VCAN^+^, CD300E^+^, CD163^low^, MRC1^low^), and dendritic cells (DCs; CD1C^+^, CLEC10A^+^) (Figure 3e; Figure S3c). As shown in Figure S3f, the proportions of these cell clusters showed significant variation among the samples. For TAMs, the major components of myeloid cells, SPP1^high^ TREM2^low^ TAMs and SPP1^low^ TREM2^high^ TAMs, were enriched in PSCC, whereas SPP1^low^ TREM2^low^ TAMs and CD209^+^ TAMs were enriched in adjacent normal tissue. Macrophages are extremely heterogeneous populations that have a combination of proinflammatory and anti‐inflammatory functions. The two extremes in the spectrum of macrophage function are represented by the M1 and the M2 phenotypes [23]. We calculated the M1/M2 scores of each macrophage subtype. SPP1^high^ TREM2^low^ exhibited more M2‐like features, while CD209^+^ TAMs displayed more M1‐like features (Figure S3d). To improve the understanding of the function of TAMs, we calculated the angiogenesis and phagocytosis scores of TAMs and found significant heterogeneity among them (Materials and Methods). SPP1^high^ TREM2^low^ TAMs scored higher in angiogenesis than the other TAMs, while CD209^+^ TAMs had the most obvious phagocytosis ability (Figure 3g). Given those results and their high abundance in tumors, we concluded that SPP1^high^ TREM2^low^ TAMs act as a protumor subtype in PSCC, which is consistent with the recent report that SPP1^+^ TAMs are associated with angiogenesis and tumor metastasis [24]. In addition, CD209^+^ TAMs exhibited a relatively higher phagocytosis score and a lower angiogenesis score and were found to be less abundant in PSCC. This observation may suggest a potential antitumor role for these cells in the PSCC microenvironment. Finally, through comprehensive cell‐cell communication analysis, we identified the NECTIN2‐TIGIT axis as a notably strong ligand‐receptor interaction between SPP1^low^ TREM2^high^ TAMs and exhausted T cells. Given the known association of TIGIT with T cell exhaustion, this interaction emerged as a particularly interesting candidate among the various predicted interactions observed in tumor samples (Figure 3h,i) [25]. To further support this interaction, we performed multiplex immunofluorescence staining, which revealed close co‐localization of NECTIN2^+^ TAMs and TIGIT^+^ CD4^+^ T cells in tumor tissue (Figure S3e), providing direct visual evidence of the spatial proximity between ligand‐ and receptor‐expressing cells. This interaction might be a crucial mechanism by which tumor‐associated macrophages facilitate the process of T cell exhaustion.

### 2.4 CAF Subpopulations Have Close Crosstalk with Malignant Cells

**FIGURE 4:**
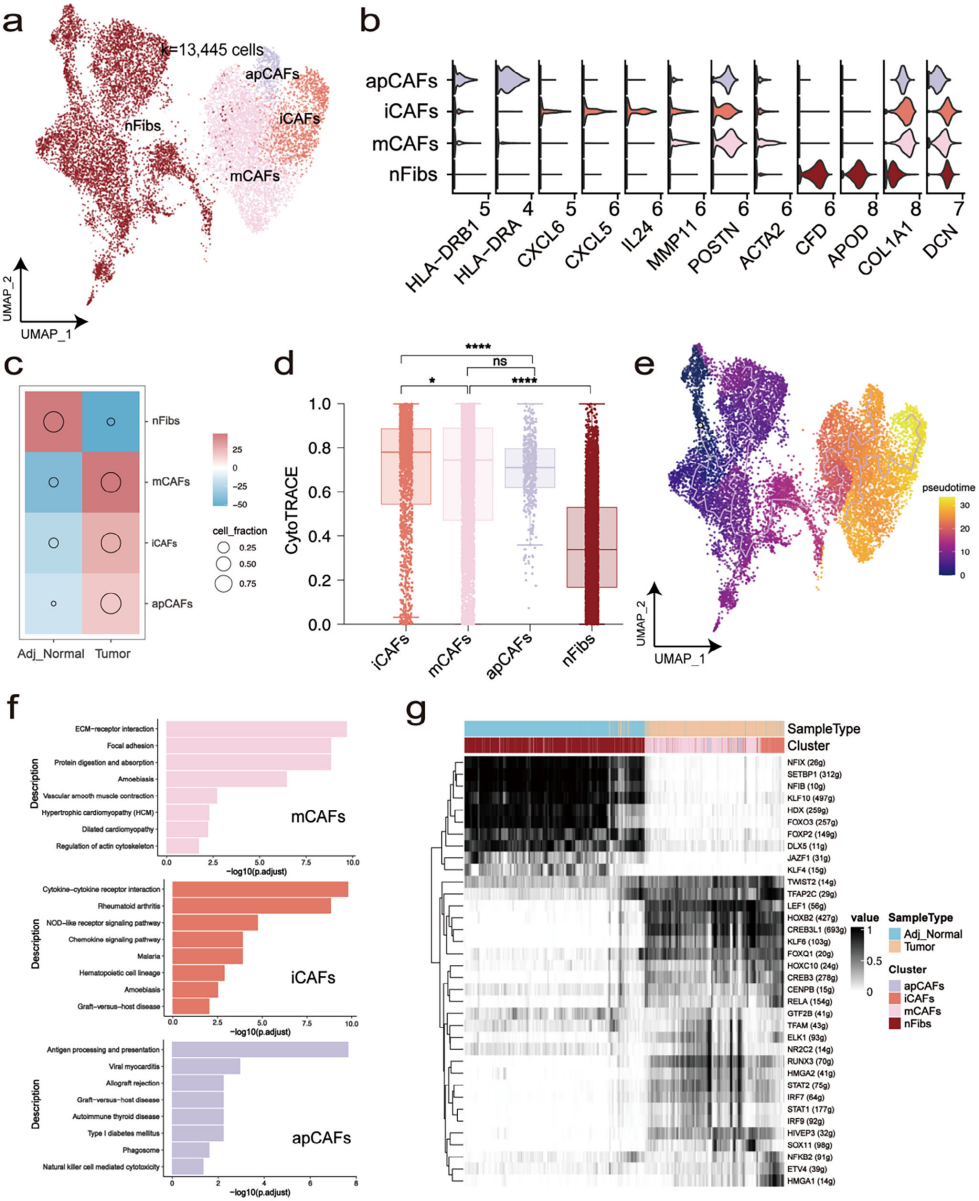
Characterization of specific interaction for fibroblast. (a) UMAP of different subsets in fibroblast. (b) Violin plot exhibiting the expression distribution of signature genes of fibroblast among different subsets. Expression values were computed using Seurat's NormalizeData() function with log1p transformation. (c) Pearson residuals and cell fraction of each cell cluster in different sample types. (d) Distribution of CytoTRACE score of each cell type, ranking by the median value. (e) Trajectory of fibroblast by Monocle3 analysis. (f) The enriched GSEA pathway of iCAFs and the GO terms of mCAFs when tumor samples compared with adjacent normal samples. (g) Binary regulon activity matrix of TFs predicted by SCENIC.

CAFs have been reported to promote tumor cell proliferation, contribute to therapy resistance, and facilitate immune exclusion by secreting growth factors, inflammatory ligands, ECM proteins and other factors [26, 27]. Therefore, we focused on fibroblasts and investigated their complexity and dynamic relationships within the TME. A total of 13 445 fibroblasts were reclustered into four subtypes: normal fibroblasts (nFibs), matrix‐related cancer‐associated fibroblasts (mCAFs), inflammatory cancer‐associated fibroblasts (iCAFs) and antigen‐presenting cancer‐associated fibroblasts (apCAFs) (Figure 4a,b). Remarkably, mCAFs, iCAFs and apCAFs were enriched in tumor (Figure 4c). CytoTRACE and Monocle3 results both revealed that nFibs were prone to differentiate into mCAFs, iCAFs and apCAFs (Figure 4d,e). To evaluate the concordance between Monocle3 and CytoTRACE, we correlated pseudotime values inferred by Monocle3 with differentiation scores from CytoTRACE (Figure S4b). A moderate but significant positive correlation was observed (Pearson's R = 0.54, *p* < 0.001), indicating that both methods captured a comparable direction of cellular progression. We identified upregulated functional pathways in tumor samples vs. adjacent normal samples. Focal adhesion and ECM‐associated pathways were significantly enriched in mCAFs, which was consistent with a previous in vitro study (Figure 4f) [28]. Several inflammation‐associated pathways were enriched in iCAFs, such as “Cytokine−cytokine receptor interaction” and “Chemokine signaling pathway”. Next, we investigated the upregulated transcripts in fibroblasts and found that LEF1 and CREB3L1 were upregulated in mCAFs (Figure 4g), both of which have been shown to regulate tumor progression and metastasis by promoting ECM signaling activation [29, 30]. In iCAFs, we observed a significant increase in the expression of HMGA1 and ETV4. Overexpression of HMGA1 has been linked to elevated cyclooxygenase‐2 (COX‐2) [31], while ETV4 contributes to liver inflammation and the occurrence of HCC by activating the transcription of TNF‐α and MAPK11 [32]. To further explore the relationships between CAFs and tumor cells, we performed cell‒cell communication analysis using CellChat [33]. Surprisingly, iCAFs and mCAFs had the strongest crosstalk with tumor cells, especially with EMT‐Mal, mediated through multiple signaling pathways, including FN1 and THY1 signalling pathway (Figure S4c,d), which are known to promote EMT [34, 35]. Interestingly, the main receptors in tumor cells, particularly for SEMA3C^high^ Mals, were integrins, capable of recognizing these ligands. As cell adhesion receptors, integrins regulate various cellular functions crucial for tumor initiation, progression, and metastasis [36]. In summary, iCAFs and mCAFs might promote the EMT of PSCC cells by cell‒cell communication through various signalling pathways.

### 2.5 POSTN^+^ Pericytes Promote the Angiogenesis and ECM Remodeling in PSCC

**FIGURE 5:**
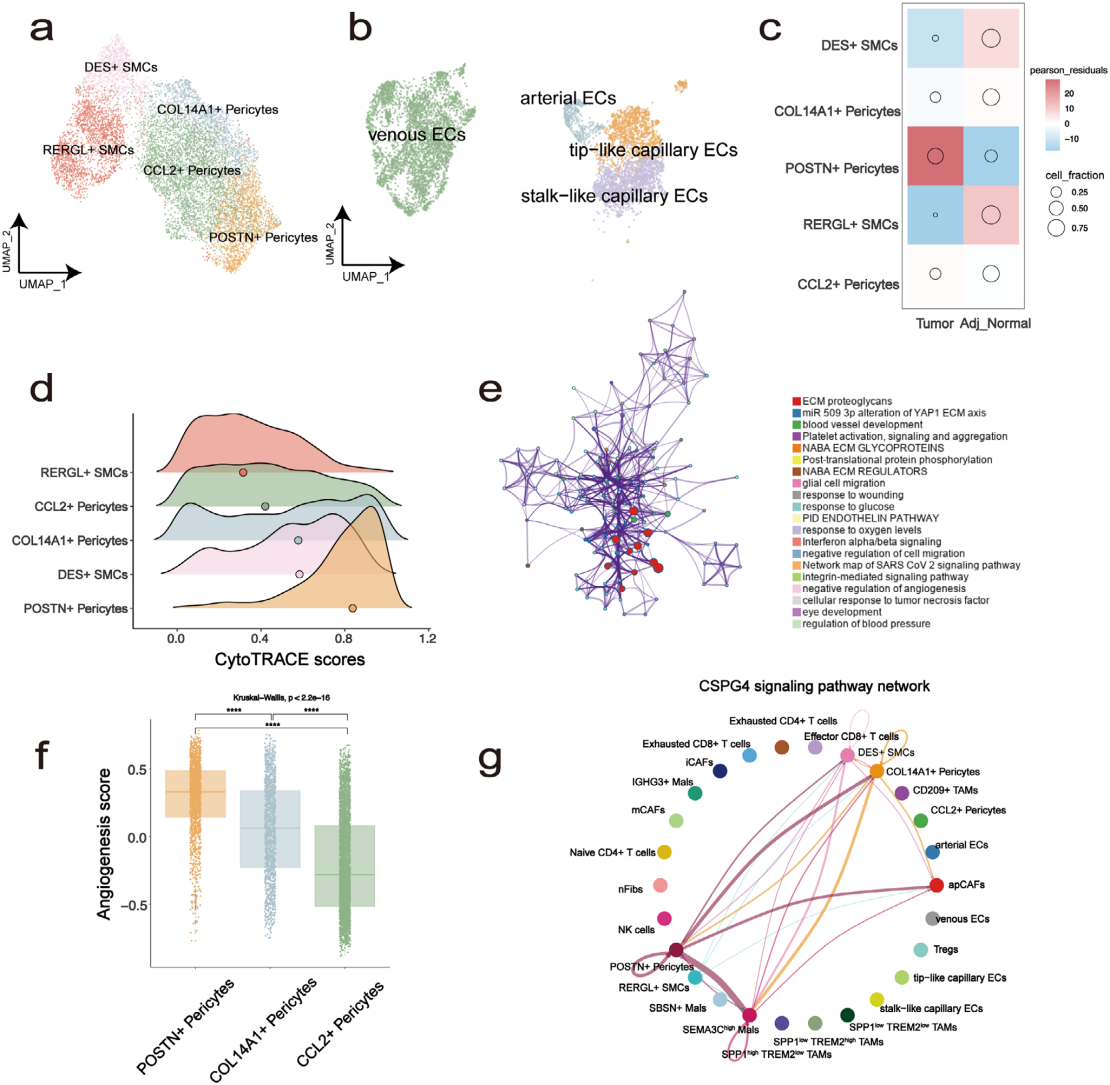
Assessment of TME's changes in endothelial cell and pericytes/SMCs. (a) UMAP of different subsets in pericytes/SMCs. (b) UMAP of different subsets in endothelial cell. (c) Pearson residuals and cell fraction of each cell cluster in different sample types. (d) Distribution of CytoTRACE score of each cell type, ranking by the median value. (e) Enrichment analysis of DEGs in POSTN^+^ pericytes by Metascape, based on differentially expressed genes between POSTN^+^ pericytes and the other two pericyte subsets. Node size reflects the number of genes associated with each enriched term; Node color represents manually curated functional categories or pathways, as indicated in the accompanying legend; Edge thickness denotes the degree of gene overlap between terms; *p* value < 0.05. (f) Comparison of angiogenesis score among three kinds of pericytes, ranking by the median value. (g) Cellular interactions of CSPG4 signalling pathway network in PSCC.

Vascular invasion is a significant predictor of PSCC progression [37]. Next, we systematically analysed ECs and pericytes/SMCs, which could be classified into the following subgroups: CCL2^+^ pericytes (RGS5^+^, CCL2^high^, MYH11^+^, POSTN^−^), POSTN^+^ pericytes (RGS5^+^, POSTN ^+^, COL1A1^high^, COL3A1^high^), COL14A1^+^ pericytes (RGS5^+^, COL14A1^high^, MYH11^+^, POSTN^−^), RERGL^+^ SMCs (ACTA2^+^, RERGL^+^), DES^+^ SMCs (ACTA2^+^, DES^+^), arterial ECs (SEMA3G^+^, HEY1^+^), tip‐like capillary ECs (RGCC^+^, PLVAP^+^, APLN^+^), stalk‐like capillary ECs (PLVAP^+^, ACKR1^+^, TSPAN7^+^) and venous ECs (ACKR1^+^, SELE^+^, IL6^+^) (Figure 5a; FigureS5a). When comparing cellular abundances across different samples, we found that POSTN^+^ pericytes and tip‐like capillary ECs were the most prominent subpopulations (Figure 5b; Figure S5b). The CytoTRACE results showed that POSTN^+^ pericytes had the most stem cell‐like features (Figure 5c), implying that they are in a very active state within the TME. Functional enrichment analysis was performed on the differentially expressed genes between POSTN^+^ pericytes and the other two pericyte subsets. The enriched pathways included blood vessel development and extracellular matrix (ECM) organization (Materials and Methods, Figure 5e), which are closely associated with tumor progression and therapeutic resistance [38]. Next, we collected a set of genes associated with pericyte‐mediated angiogenesis to evaluate the angiogenic potential across pericyte subsets. Among the three identified subtypes, POSTN^+^ pericytes exhibited the highest angiogenic signature scores, suggesting a prominent role in vascular remodeling [39]. Cell‐cell interaction analysis showed strong interactions between POSTN^+^ pericytes and SEMA3C^high^ Mals via the CSPG4 signalling pathway in tumor samples, with no detectable interaction with other malignant cell subsets(Figure 5g; Figure S5c). These findings suggest a preferential signaling bias from POSTN^+^ pericytes toward SEMA3C^high^ tumor cells, which may constitute a putative functional axis. A previous study found that the overexpression of CSPG4 could worsen prognosis by increasing tumor initiation, growth rates, neovascularization, and cellular proliferation [40]. Consistently, multiplex immunofluorescence staining revealed that POSTN^+^ pericytes were spatially adjacent to CD31^+^ endothelial structures in tumor tissues (Figure S5d), supporting their localization within angiogenic niches. Therefore, the close interactions between POSTN^+^ pericytes and tumor cells may be a key factor driving PSCC progression.

### 2.6 SEMA3C is an Effective Biomarker of PSCC Progression

**FIGURE 6:**
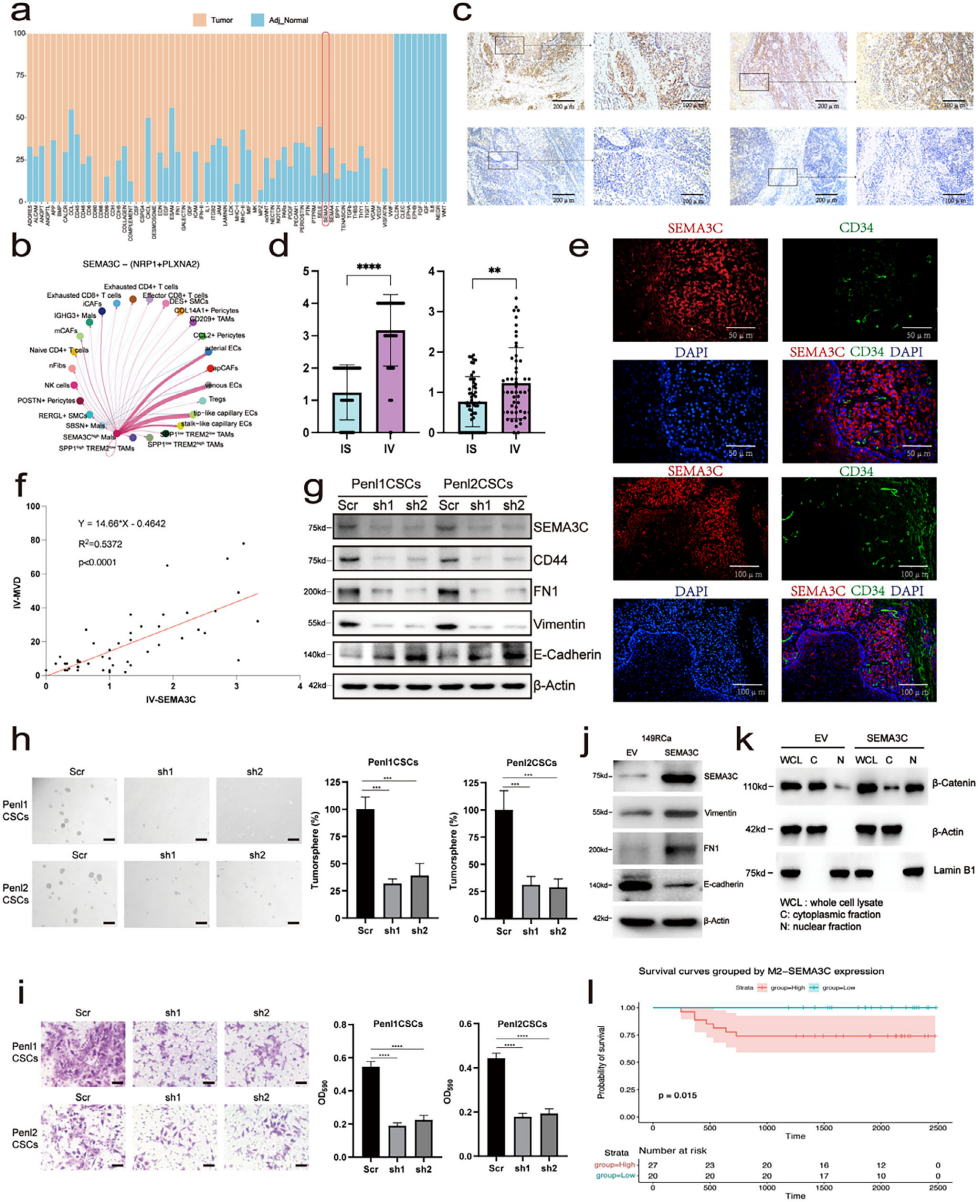
SEMA3C signaling promotes stemness, EMT and angiogenesis in PSCC. (a) Based‐on cell interaction difference between tumor and adjacent normal samples, the signalling pathways were ranked. (b) Cellular interactions of SEMA3C signalling pathway network in PSCC. (c) IHC staining of SEMA3C in PSCC tissues. (d) The picture in the left is the immunofluorescence staining intensity of SEMA3C, while the picture in the right is the expression score of SEMA3C, *n* = 47. (e) Immunofluorescence co‐staining of human PSCC tissue (20x is positioned above, while 40x is positioned below). DAPI (blue), CD34 (green), SEMA3C (red), in individual and merged channels are shown. Experiment was performed in two independent patients. (f) Scatter plot of MVD in IV and the expression score of SEMA3C in tumor samples. Different dots represent different samples, *n* = 47. (g) Western blot analysis of stemness‐associated genes and EMT‐related genes expression following shRNA‐mediated knockdown of SEMA3C in Penl1 and Penl2 cells. (h) Tumorsphere formation assay in Penl1CSCs and Penl2CSCs following shRNA‐mediated knockdown of SEMA3C (sh1 and sh2). Representative bright‐field images (left) and quantification of tumorsphere formation efficiency (right) are shown. Scale bar, 500 µm. (i) Matrigel‐based transwell invasion assay performed in Penl1CSCs and Penl2CSCs following shRNA‐mediated knockdown of SEMA3C. Left: representative images of crystal violet‐stained invaded cells. Right: quantification of OD_590_ absorbance as a proxy for invasive cell number. Scale bar, 100 µm. Statistical significance was assessed using unpaired two‐tailed t‐tests; ^***^ *p* < 0.001, ^****^ *p* < 0.0001. (j) Western blot analysis of EMT markers in 149Rca cells transfected with empty vector (EV) or SEMA3C. β‐Actin was used as a loading control. (k) Western blot analysis of β‐Catenin localization in cytoplasmic (C) and nuclear (N) fractions from 149Rca cells expressing EV or SEMA3C. Whole cell lysate (WCL) was included for reference. SEMA3C overexpression promoted β‐Catenin nuclear translocation. β‐Actin and Lamin B1 served as cytoplasmic and nuclear markers, respectively. (l) Kaplan‐Meier survival curves comparing overall survival between patients with high and low IV‐SEMA3C expression. Patients were stratified into high (red) and low (blue) expression groups based on median IV‐SEMA3C scores. Shaded areas indicate 95% confidence intervals. The table below shows the number of patients at risk at each time point, *n* = 47.

Recent studies have emphasized the importance of communication between tumor cells and other cells in the TME [41, 42]. To identify the key interactions mediating TME reprogramming in PSCC, we conducted cell‐cell communication analysis using both tumor and adjacent normal tissue samples. The most prominent signaling pathways, ranked by interaction abundance, were identified as specifically existing in the tumor or adjacent normal samples (Figure 6a). Among them, the SEMA3 pathway was found to connect SEMA3C^high^ Mals to a wide range of other cells in tumor samples, especially tip‐like capillary ECs (Figure 6b; Figure S6a). Next, we performed immunohistochemistry (IHC) staining in a clinical cohort comprising 47 patients (Table S2) to validate the expression of SEMA3C in PSCC. We found that in most samples, positive SEMA3C staining tended to be located within infiltrating tumor foci (IV, Figure 6c), and SEMA3C expression was obviously upregulated in the IV area compared to the IS area (Figure 6d).

We noticed that a receptor expressed by capillary ECs could specifically recognize SEMA3C, which was highly expressed in SEMA3C^high^ Mals, so we performed immunofluorescence costaining of SEMA3C (representing SEMA3C^high^ Mals) and CD34 (the canonical marker of vascular ECs) to validate the crosstalk. Immunofluorescence labeling demonstrated the proximity of CD34‐positive and SEMA3C‐positive cells in PSCC tissue (Figure 6e), implying a potential interaction between capillary ECs and SEMA3C^high^ Mals. In addition, the MVD and expression of SEMA3C within the IV region was significantly positively correlated (Figure 6f), suggesting that SEMA3C promotes angiogenesis [43]. Furthermore, we evaluated the association between SEMA3C expression and TNM stages and found significantly higher expression of SEMA3C in stage III and IV samples than in stages I and II samples (Figure S7a). Notably, SEMA3C expression in IV region is significantly higher than IS region in Phases III and IV samples (Figure S7b). Next, we explored the expression difference of SEMA3C among samples with different degrees of lymph node metastasis and found that SEMA3C expression was higher in N3 and N4 samples than in N1 and N2 samples, and SEMA3C expression was mainly found in the IV area (Figure S7c,d). The above results indicate that SEMA3C is correlated with the progression and metastasis of PSCC. Therefore, we proposed that SEMA3C may regulate the expansion/invasion phenotypes in PSCC. Penl1 and Penl2 cells were transduced with non‐targeting scramble (Scr) or 2 shRNAs targeting SEMA3C (sh‐1 and sh‐2). As shown in Figure 6k, SEMA3C expression was significantly reduced in Penl1 and Penl2 cells transduced with the 2 shSEMA3C lentiviruses, whereas it was not significantly affected by Scr shRNA (Materials and Methods). Meanwhile, this knockout resulted in a simultaneous decrease in the expression levels of CD44, VIM, and FN1, while E‐Cadherin expression increased (Figure 6g). The reduction in CD44 suggests a decrease in tumor cell stemness, while the changes in VIM, FN1, and E‐Cadherin indicate a reduction in EMT within the tumor cells. The tumor sphere‐forming capacity of cell with SEMA3C knockout was significantly reduced, indicating that SEMA3C plays a crucial role in tumor progression (Figure 6h). To access the effect of SEMA3C on cancer cell invasion in vitro, we performed a Transwell invasion assay (Materials and Methods). Notably, the invasion ability was significantly reduced in the SEMA3C knockdown groups (Figure 6i). To assess the functional role of SEMA3C, we overexpressed SEMA3C in 149Rca cells. This promoted cell proliferation, migration, and invasion (Figure S7e–g), accompanied by reduced E‐cadherin expression and nuclear translocation of β‐Catenin (Figure S7h). Western blot confirmed increased Vimentin and FN1 and decreased E‐cadherin levels (Figure 6j), along with β‐Catenin nuclear accumulation (Figure 6k), indicating activation of EMT and Wnt signaling pathways. Finally, we performed survival analysis using the same patient cohort stratified by SEMA3C expression levels. Patients with high SEMA3C expression exhibited significantly poorer overall survival than those with low expression (*p* = 0.015; Figure 6l), highlighting its potential as a prognostic indicator in PSCC. These findings suggest that SEMA3C not only correlates with aggressive tumor phenotypes but also functionally promotes proliferation, invasion, and angiogenesis in PSCC. Collectively, SEMA3C may serve as a functional driver of tumor progression and a potential prognostic biomarker in advanced disease.

## 3 Materials and Methods

### 3.1 Patients and Samples Collection

Nine tumor samples and six adjacent normal tissues (approximately 2 cm from the tumor margin) were collected from the foreskin region of ten patients with a confirmed pathological diagnosis of penile squamous cell carcinoma (PSCC). All patients were from Chongqing University Three Gorges Hospital and signed an informed consent form for providing samples. Detailed clinical characteristics and pathological information are summarized in Table S1. Our study was approved by the institutional ethics committee of Chongqing University Three Gorges Hospital (No. 2024‐KELUN‐SHEN‐193).

### 3.2 Isolation of Single Cells

Fresh samples were stored at 2°C–8°C in MACS tissue preservation solution (Miltenyi Biotec) and processed within 2–3 h after surgical resection to minimize degradation. Prior to dissociation, specimens were washed three times with Hanks' Balanced Salt Solution (HBSS). The tissues were then minced into small pieces and digested using GEXSCOPE Tissue Dissociation Solution (Singleron Biotechnologies) at 37°C for 25 min with continuous agitation. Following digestion, the cell suspension was washed with Dulbecco's Phosphate Buffered Saline (DPBS, BI) and filtered through a 40‐µm sterile strainer (Falcon). Cells were centrifuged at 2000 rpm for 10 min, and the resulting pellets were resuspended in the appropriate buffer. Finally, the cell suspensions were stained with trypan blue (Sigma, USA), and cell viability was assessed under a microscope.

### 3.3 Single‐Cell Library Preparation and Sequencing

#### 3.3.1 Singleron Platform

Utilizing the Singleron Matrix Single Cell Processing System, single‐cell suspensions (1105 cells/mL) in PBS (HyClone) were loaded into microfluidic devices (Singleron). The procedures that followed were carried out in accordance with the manufacturer's guidelines [44]. With 150 bp paired end reads, pools were finally sequenced on the Illumina HiSeq X.

#### 3.3.2 BD Rhapsody Platform

A 400 µL single‐cell suspension with cold Sample Buffer (BD Biosciences) and cells obtained was put onto a BD Rhapsody cartridge with more than 200 000 microwells. As directed by the BD RhapsodyTM Single cell 3' whole transcriptome amplification (WTA) with sample multiplexing kit technique, the following procedures were carried out (BD Biosciences). The final libraries were sequenced using 150 bp paired end reads on an Illumina HiSeq X.

#### 3.3.3 SeekOne DD Chip Platform

The tumor tissues were dissociated using the Multi‐Tissue Dissociation Kit 2 (Miltenyi) after being washed with ice‐cold RPMI 1640. DNase treatment was carried out according to the manufacturer's instructions. Erythrocytes were removed using Miltenyi's erythrocyte depletion reagent (130‐094‐183), and cell count and viability were assessed using the Countstar Rigel S2 Cell Analyzer with AO/PI staining. The removal of debris and dead cells was performed based on the experimental requirements, using Miltenyi's removal kits (130‐109‐398/130‐090‐101). Finally, the cells were washed twice with RPMI 1640 and resuspended in 1 × PBS with 0.04% bovine serum albumin at a concentration of 1 × 10^6 cells per mL.

### 3.4 Raw Data Processing

#### 3.4.1 For Data Generated with Singleron Platform

Reads were mapped to the human genome (GRCh38) using scopetools (https://github.com/SingleronBio/SCOPE‐tools). After filtering read one based on the sequence information, the cell barcode and unique molecular identifier (UMI) were first extracted, and then the corrected barcode and original UMI sequence were added to the ID of read two. Read two was then chopped by cutadapt, and STAR was used to align it to the reference genome [45, 46]. Additionally, featureCounts was applied to analyze target reads for genes' genomic positions (ensemble version 99) [47]. Finally, reads with the same cell barcode, UMI and gene were grouped together to count the number of UMIs per gene per cell.

#### 3.4.2 For Data Generated with BD Platform

The BD Rhapsody Analysis pipeline (BD Biosciences), which was integrated into the CWL‐runner, was used to process the FASTQ files. Shortly, low‐quality read pairs were eliminated and the quality‐filtered R1 reads were analyzed to identify the cell label and UMI sequences. After that, STAR was used in the pipeline to map the filtered R2 reads to the transcriptome. Reads having the same gene, cell label, and UMI sequence were combined into a single raw molecule. Recursive substitution error correction (RSEC), an error correction technique created by BD Biosciences, was used to correct the acquired counts for sequencing and PCR errors. Using barcoded oligo‐conjugated antibodies (single‐cell multiplexing kit; BD Biosciences), the BD Rhapsody Analysis pipeline inferred the sample's origin.

#### 3.4.3 For Data Generated with the SeekOne DD Chip Platform

The raw reads were processed using the SeekOneTools (Beijing SeekGene BioSciences). All reads were aligned against the reference genome GRCh38 (ensemble version 99).

### 3.5 Quality Control, Batch Correction, Clustering

The UMI count data of each sample was imported into Seurat to process and analyze (V4.1.0). For quality control, cells meeting one of the following thresholds were excluded: 1) gene count less than 200 or more than 4 000 gene; 2) UMI count more than 20 000; 3) mitochondrial content > 20%. In order to remove potential doublets, we used the default options of the Scrublet software and possible doublets were found and eliminated [48]. Then single‐cell gene expression data was normalized with a scale factor of 10 000 by “NormalizeData” functions of Seurat. The top 2000 most variable genes were selected out using the “FindVariableGenes” and the range of expression values for each gene was standardized using a linear scaling technique. To reduce the dimensionality of scRNA‐seq data, principal component analysis (PCA) was performed, and top 50 PCs were used for downstream analysis with “Elbowplot” function. Since samples were from diverse patients and platforms, we used harmony and supplied sampleID and platform as two technical factors for batch correction [18]. The main cell clusters were identified with the “FindClusters” function, with the resolution of 0.25.

### 3.6 Cell‐Type Annotation

Using the gene expression of canonical markers, we have annotated these main cell types: vascular EC (PECAM1, VWF, PLVAP, ACKR1); fibroblast (COL1A1, DCN, LUM); pericyte/SMC (RGS5, ACTA2); myeloid cell (LYZ, HLA‐DRA, CD14); T cell (CD2, CD3D, TRBC2, TRBC1); epithelial cell (KRT5, KRT14, KRT17); neutrophil (CSF3R, FCGR3B); proliferative cell (TOP2A, MK167); B cell (IGHA1, JCHAIN, MS4A1); mast cell (IL1RL1, TPSB2, TPSAB1); lymphatic EC (PECAM1, LYVE1);malignant cell (COL17A1, PTHLH, ITGB1, DST). To distinguish malignant epithelial cells from normal epithelial populations, we applied the inferCNV workflow (https://github.com/broadinstitute/inferCNV), which infers large‐scale chromosomal copy number variations from scRNA‐seq data. Normal fibroblasts and endothelial cells were used as reference cell populations to establish a baseline for CNV inference.

### 3.7 Differentially Expressed Genes Analysis

Differentially expressed genes (DEGs) of the cell types were identified by “FindMarkers” function of Seurat. Next, genes with *p*_val_adj < 0.05, avg_log2FC > 1 were defined as up‐regulated genes and genes with *p*_val_adj < 0.05, avg_log2FC < ‐1 were defined as down‐regulated genes.

### 3.8 Gene Functional Annotation

To describe the function features of cell subtypes, we used the clusterProfiler (V4.1.4) package [49], which was applied for Gene Ontology (GO) and Kyoto Encyclopedia of Genes and Genomes (KEGG) pathway analysis with significant DEGs. Pathways with *q* value < 0.05 were regarded as significant enriched results. In additional, POSTN^+^ pericytes was picked out to calculate the difference genes with EC and Fibroblast respectively. The calculated differential genes from the two groups were taken as intersection to be put into Metascape (www.metascape.org) to investigate the function of POSTN^+^ pericytes [50].

### 3.9 Gene Set‐Based Signature Scoring

We collected GO terms mapping to the hallmarks of cancer [51], which were used to evaluate the malignant cell. The epithelial‐mesenchymal transition (EMT) signature, PI3K signaling pathway genes, and p53 signaling pathway genes were downloaded from the Molecular Signatures Database (MSigDB), and were used to evaluate the corresponding pathway activity scores of malignant cells. We evaluated the phagocytosis score of myeloid cells by collecting its associated genes from KEGG (hsa04666) and the angiogenesis score of myeloid by downloading angiogenesis‐related gene set from MSigDB. To evaluate the exhaustion status of T cell in our study, we used a group of exhaustion‐related genes to define the exhaustion score. Specifically, the exhaustion score was defined as the average expression of TIGIT, LAG3, CTLA4, RBPJ, MYO7A, TIM3, GZMB, VDR, ZBED2, PRDM1 and IFI16 [52].

The score of above‐mentioned pathways or signature was all calculated using GSVA R package (V1.40.1) [53]. To compare the differences of scores between different sample or cell types, we used the Wilcoxon signed‐rank test that implemented in the ggpubr package (V0.4.0) to perform significance tests.

### 3.10 CytoTRACE Analysis

To map cell differentiation character in the TME, pseudotime analysis was performed with CytoTRACE analysis [15]. We performed CytoTRACE analysis with default parameters following the official guidance, which could predict cell differentiation states from scRNA‐seq data.

### 3.11 Pseudotime Analysis

Pseudotime analysis was performed using Monocle3 [54, 55]. Seurat objects were converted to Monocle3 cell_data_set() format using as.cell_data_set(). UMAP coordinates were transferred from Seurat. Cells were clustered with cluster_cells(), and trajectories were learned using learn_graph(). Cells were ordered in pseudotime with order_cells(), and the root state was defined based on known marker gene expression.

### 3.12 Cross‐Cancer Comparative Analysis

To investigate the transcriptional similarity of key TME cell subsets across squamous cell carcinomas (SCCs), we utilized published scRNA‐seq datasets of cutaneous(CSCC), cervical (CESC), laryngeal (LSCC), oral (OSCC), and lung (LUSC) squamous cell carcinomas generated in our previous studies [38].

T cells, TAMs, and CAFs from each cancer type were extracted based on canonical markers. We performed integration and similarity analysis using Seurat v4.1.0 with default settings. Transcriptomic similarity matrices were computed based on the average gene expression profiles using Pearson correlation and visualized with hierarchical clustering.

### 3.13 Survival Analysis

Survival analysis was performed using bulk transcriptomic and clinical data from a PSCC patient cohort (*n* = 47). Patients were stratified into SEMA3C‐high and SEMA3C‐low groups based on the median expression value. Kaplan‐Meier survival curves were generated using the survminer and survival packages in R (v4.1.0), and statistical significance was determined by log‐rank test.

### 3.14 Comparison of Cell‐Type Proportions Using Pearson Residuals

To assess differences in cell‐type proportions between tumor and adjacent normal samples, we first constructed a contingency table of observed cell counts for each sample type and cell type.

Pearson residuals were then manually calculated for each group‐cell type pair using the classical formula:

$$\mathrm{Residual}=\left(\mathrm{Observed}-\mathrm{Expected}\right)/\sqrt{\mathrm{Expected}}$$

The expected count was calculated as:

$$\mathrm{Expected}=\left(\mathrm{Row}\;\mathrm{total}\times \mathrm{Column}\;\mathrm{total}\right)/\mathrm{Grand}\;\mathrm{total}$$

Positive residuals indicate that a given cell type was overrepresented in a group, while negative residuals indicate underrepresentation.

### 3.15 Cell‐to‐Cell Interactions

CellChat (V1.1.3, https://github.com/sqjin/CellChat) is a powerful tool that is able to quantitively infer and assess intercellular communication networks from scRNA‐seq data [33]. Through network analysis and pattern recognition approaches, we used CellChat (version 1.0.0) to anticipate the major signalling inputs and outputs for cells as well as how those cells and signals collaborated to perform certain functions. Furthermore, we compare the results between tumor samples and adjacent normal samples. We primarily used the “netVisual aggregate” function to illustrate the specific differences in interactions between the two sample types (tumor sample, adjacent normal samples) and we used the “plotGeneExpression” function to highlight the degree to which the genes encoding ligands and receptors were expressed. All cell interaction visualizations were plotted using the CellChat package.

### 3.16 Statistical Analysis

All statistical analyses were performed using R (version 4.2.1) and relevant R packages. For differential gene expression (DEG) analysis, we used the FindMarkers() function in Seurat (v4.1.0), which applies a Wilcoxon ranksum test by default. Genes with an adjusted *p*‐value (Benjamini‐Hochberg method) < 0.05 and |log_2_ fold change| > 1 were considered significantly differentially expressed.

For signature scoring, pathway activity scores were calculated using the GSVA package (v1.40.1), and group comparisons were performed using the Wilcoxon signed‐rank test, as implemented in the ggpubr package (v0.4.0).

For functional enrichment analysis (GO, KEGG), the clusterProfiler package (v4.1.4) was used, and pathways with a *p*‐value < 0.05 (Benjamini‐Hochberg corrected) were considered significantly enriched.

Cell‐type proportion differences were assessed by manually calculating Pearson residuals based on contingency tables, using the classical formula:

$$\mathrm{Residual}=\left(\mathrm{Observed}-\mathrm{Expected}\right)/\sqrt{\mathrm{Expected}}$$

$$\mathrm{Expected}=\left(\mathrm{Row}\;\mathrm{total}\times \mathrm{Column}\;\mathrm{total}\right)/\mathrm{Grand}\;\mathrm{total}$$

Unless otherwise stated, all statistical tests were two‐sided, and results were considered statistically significant when the adjusted *p*‐value < 0.05. Data visualization was performed using ggplot2, ComplexHeatmap, and GraphPad Prism.

### 3.17 Immunofluorescence Costaining

Costaining was performed to show colocalization of the signals of specific cells. After permeabilization with 0.4% Triton X‐100 for 10 min, cytospun cells were fixed with 4% neutral buffered formalin for 15 min. After 15 min in Background Sniper (Biocare Medical LLC, Pacheco, CA, USA), the cells were next treated for 30 min with 5% bovine serum albumin (BSA) (Sigma–Aldrich) to prevent background. The cells were then treated with primary antibodies for an overnight period and secondary antibodies for 2 h. In different groups, the anti‐SEMA3C antibody (affinity biosciences) was used to be primary antibody to detect the SEMA3C protein (shown in red). The corresponding secondary antibodies were the anti‐Vimentin antibody (Beyotime biotech Inc), the anti‐ Ecadherin antibody (Beyotime biotech Inc), the anti‐CD34 antibody (zymed laboratories Inc) (show in green), respectively. Slides were stained with diamidino phenyl indole (DAPI) for the nucleus and then covered with Mowiol mounting solution and cover slipped. A 3DHISTECH Scan II Fluorescence Slide Scanner was used to scan cells at a 20x magnification (40 0.27 m resolution), and CaseViewer 2.4 was used to gather and view the images (3DHISTECH, Budapest, Hungary). Finally, representative images from at least three biological replicates are shown.

### 3.18 Immunohistochemistry

Sections of 4 um thickness were cut from paraffin‐embedded formalin‐fixed specimens from PSCC patients. Tissue slices were heated to 65°C in an oven for 2 h, then deparaffinized for 40 min before being rehydrated with varying alcohol concentrations. The deparaffinized pieces were cooked in a microwave for 20 min on low power while submerged in 10 nm citric acid that was already boiling (pH 6.0). The subsequent 15‐min incubation in 150 mm Tris‐HCl at pH 9 and 70°C improved antigen retrieval. After the sections had reached room temperature, they were cleaned with PBS, ringed with an immunohistochemical pen (made by Zhongshan Jinqiao Biotechnology) and sealed with peroxidase for 10 min. The goat serum (Zhongshan Jinqiao Biotechnology) was used to seal the sections after washing them with PBS and discarding the peroxidase. The slides were then treated with a primary rabbit monoclonal anti‐SEMA3C antibody at 4°C for an overnight period (1:100, affinity biosciences). The slides were incubated with IHC enhancer the following day for 20 min, and then at 37°C for 1 h with the corresponding secondary antibody conjugated with horseradish peroxidase. Following the addition of the DAB (3,3‐diaminodbenzidine) substrate, the sample was counterstained for 5 min with hematoxylin. Finally, the sections were dehydrated, cleared, and mounted in aqueous mounting medium for microscopic evaluation. Images shown are representative of at least three independent biological replicates.

### 3.19 Antibodies

The following primary and secondary antibodies were used in this study. For immunofluorescence staining, primary antibodies included anti‐SEMA3C (rabbit, Affinity Biosciences, 1:100), anti‐Vimentin (mouse, Beyotime, 1:100), anti‐E‐cadherin (mouse, Beyotime, 1:200), anti‐β‐Catenin (rabbit, [Vendor], 1:200), anti‐CD34 (mouse, Zymed Laboratories Inc, 1:100), anti‐POSTN (rabbit, Proteintech, 1:100), anti‐TIGIT (rabbit, Abcam, 1:100), and anti‐NECTIN‐2 (rabbit, Abcam, 1:100). Secondary antibodies included Alexa Fluor 488 goat anti‐mouse IgG and Alexa Fluor 594 goat anti‐rabbit IgG (Invitrogen, 1:500). Nuclei were counterstained with DAPI.

For immunohistochemistry (IHC), rabbit monoclonal anti‐SEMA3C antibody (Affinity Biosciences, 1:100) was used, followed by HRP‐conjugated goat anti‐rabbit IgG (ZSGB‐Bio, 1:200).

For Western blotting, the antibodies used were as follows: anti‐SEMA3C (rabbit, Affinity Biosciences, 1:1000), anti‐Vimentin (mouse, Beyotime, 1:1000), anti‐FN1 (rabbit, [Vendor], 1:1000), anti‐E‐cadherin (mouse, Beyotime, 1:1000), anti‐β‐Catenin (rabbit, [Vendor], 1:1000), anti‐β‐Actin (mouse, Beyotime, 1:5000), and anti‐Lamin B1 (rabbit, [Vendor], 1:1000). HRP‐conjugated goat anti‐mouse and anti‐rabbit IgG (Beyotime) were used as secondary antibodies.

All antibodies were diluted in 1% BSA or according to manufacturer instructions.

### 3.20 MVD Counting

The interstitial area with abundant microvessels adjacent to the tumor cells was examined under a 40x microscope, ensuring that the tumor necrosis and vascular sclerosis areas were excluded. The number of microvessels within five 400x fields of view were counted separately, and their average value was calculated as the microvascular density (MVD) of the specimen. Microvessels are defined as single or multiple endothelial cells, with or without lumen, and lacking smooth muscle encapsulation. Vessels were excluded from the count if they met any of the following criteria: (i) the lumen diameter exceeded the width of 8 erythrocytes; (ii) the vessel wall had a significant muscular layer; (iii) the vessel fibrosis showed signs of sclerosis; or (iv) the microvessels were necrotic.

### 3.21 Cell Lines and Sphere‐Forming Assay

Human penile cancer cell lines Penl1 and Penl2 were kindly provided by Prof. Hui Han (Department of Urology, Cancer hospital, Sun Yat‐sen University) [56].

An estimated 2000 cells were plated on a non‐treated six‐well plate (Corning Life Science, EUA) with DMEM/F12 medium supplemented with 10 ng/mL of basic fibroblast growth factor (bFGF, Lonza, Wokingham, UK), 10 ng/mL of EGF (Gibco, Carlsbad, CA, USA), and B27 supplement (1×, Gibco, Germany). After 7 days, the presence of spheres was analyzed using a phase contrast microscopy (Olympus TXK31). Spheres were defined as cell aggregates with a diameter of ≥60 µm, exhibiting regular, round morphology with clear boundaries. For each well, five randomly selected fields were imaged. Image processing and quantification were performed using a combined workflow of ImageJ and CellProfiler. The images were subjected to background subtraction and edge detection, and sphere‐like structures were identified based on area, circularity, and solidity metrics. Aggregates with irregular morphology or apparent fusion were excluded from the analysis. To ensure the robustness of the automated quantification pipeline, a subset of representative images was manually reviewed by two independent investigators. Manual counts were compared with automated counts to verify consistency. Finally, sphere formation assays were repeated in three independent experiments.

### 3.22 Western Blotting

Cell lysates were obtained using RIPA buffer. The Western blotting procedure was carried out as previously detailed [57]. Protein bands were detected with the ECL system (Abcam, Cambridge, MA, USA). At last, all experiments were performed in biological triplicates unless otherwise specified. The full, unaltered images of all Western blots are provided in Supporting Information.

### 3.23 Transwell Invasion Assay

The cell invasion assay was conducted using transwell chambers with 8 µm pores as previously described [58]. PC cells were suspended in DMEM at 5 × 10^5 cells/mL. Each transwell in a 24‐well plate was pre‐coated with 50 µL of Matrigel. The lower compartment received 600 µL of RPMI 1640 medium with 10% FBS, while 0.1 mL (0.5 × 10^5 cells) of cell suspension was added to the upper compartment. The plates were incubated at 37°C for 24 h. Invaded cells on the membrane's underside were fixed with 4% paraformaldehyde and stained with 0.2% crystal violet solution (Sigma–Aldrich, USA). After washing, the cells were imaged using an Olympus BX43 microscope. The stained cells were then eluted with 20% glacial acetic acid and quantified using an MK3 microplate reader (Thermo Scientific, USA) at 570 nm. Each condition was tested in at least three independent biological replicates.

## 4 Discussion

Postoperative recurrence and metastasis of PSCC are important factors affecting prognosis [6], and resolving the cellular heterogeneity and characteristics of the TME is beneficial for developing a more effective method to prevent the progression of PSCC. In this study, we performed scRNA‐seq analysis of 66 421 cells from 10 PSCC patients, and our results provide a new perspective regarding the heterogeneity of the TME. We uncovered key cell subsets and cellular interactions involved in PSCC pathogenesis and identified SEMA3C as a promising treatment target for clinical applications.

**FIGURE 7:**
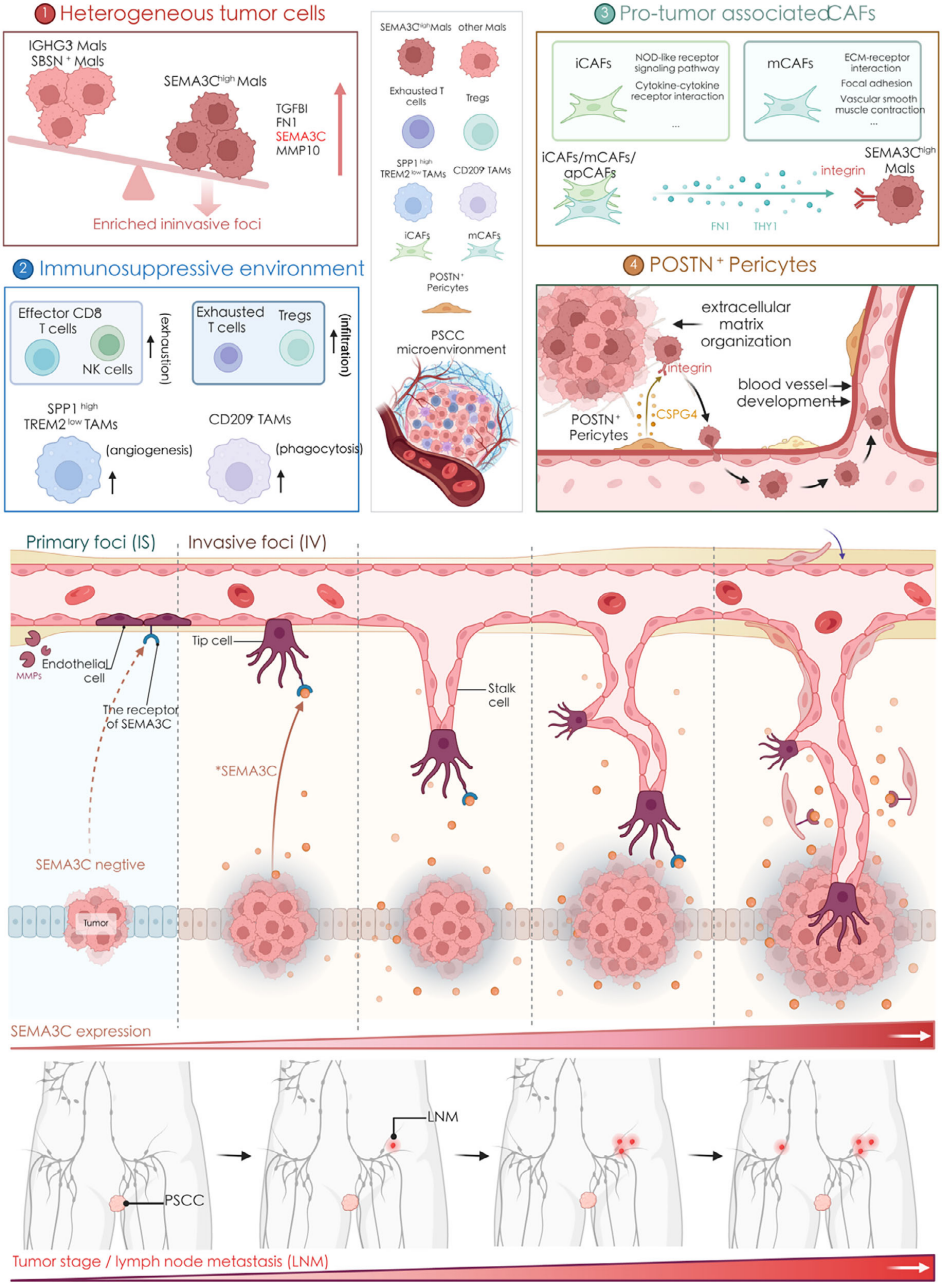
(a) Illustration showing the heterogeneity mechanism of TME during the progression of PSCC. (b) Expression level of SEMA3C influences the tumor TNM staging and the degree of lymph node metastasis by promoting angiogenesis in PSCC. The schematic illustration was created with BioRender.

Overall, we revealed heterogeneous subgroups of malignant cells. SEMA3C^high^ Mals was enriched in invasive foci and highly expressed SEMA3C and other EMT‐related genes (Figure 7a, Section 1). T cells and macrophages together generated the immunosuppressive environment of PSCC: exhausted T cells are abundant in tumor tissues, with effector T cells also being highly exhausted, and the proportion of Tregs is increased. Two heterogeneous groups of macrophages, SPP1^high^ TREM2^low^ and CD209^+^ TAMs, were enriched in PSCC and normal tissues, respectively (Figure 7a, Section 2). CAFs with distinct functions were found to interact closely with SEMA3C^high^ Mals through multiple EMT‐associated signalling pathways (Figure 7a, Section 3). T cells, TAMs, and CAFs from cutaneous, cervical, oral, laryngeal, and lung SCCs exhibited strong transcriptomic similarity to their counterparts in PSCC. Specifically, the T cells and macrophage states were highly conserved (Figure S8a), and both myofibroblastic and inflammatory CAFs from PSCC showed transcriptomic profiles similar to those in other SCCs (Figure S8b). These findings suggest that the tumor‐supportive cellular programs identified in PSCC may reflect a broader conserved TME architecture across SCCs and point to potential pan‐squamous therapeutic targets.

Notably, a specific subgroup of pericytes called POSTN^+^ pericytes, was specifically present in PSCC. Pericytes have the multidifferentiation ability of mesenchymal stem cells, allowing them to transdifferentiate into granulocytes, osteoblasts, and adipocytes [59, 60]. In addition, pericytes can transdifferentiate into (myo)fibroblasts and participate in fibrosis during kidney damage [61]. We found characteristics of angiogenesis and ECM remodeling in POSTN^+^ pericytes and revealed that they were closely linked to tumor cells; for example, they interacted with SEMA3C^high^ Mals through the CSPG4 pathway. However, the ontogeny and temporal activation of POSTN^+^ pericytes remain unclear. Whether these cells represent pre‐existing modulators within the TME or are recruited and reprogrammed in response to epithelial or vascular signals is an important question that warrants further study. Clarifying this distinction would offer critical insights into stromal plasticity and the dynamic nature of tumor‐stromal interactions in PSCC. Theoretically, ECM components can be produced by any cell, and ECM remodeling is an important protumorigenic process that promotes tumor progression [62]. Chondroitin sulphate proteoglycan 4 (CSPG4) is a cell surface proteoglycan that promotes tumor growth and metastatic dissemination by regulating cell proliferation, cell survival, drug resistance and angiogenesis [63, 64]. Higher expression of CSPG4 was associated with poorer prognosis in head and neck squamous cell carcinomas, melanoma, glioblastoma, and hepatocellular carcinoma [65, 66, 67]. Therefore, POSTN^+^ pericytes might support tumor growth and metastasis through ECM remodeling, and they may secrete CSPG4 to communicate with SEMA3C^high^ Mals and further promote angiogenesis in PSCC (Figure 7a, Section 4).

Finally, our study identified SEMA3C as a potential biomarker and functional driver of malignancy in PSCC. On the one hand, SEMA3C was specifically highly expressed in SEMA3C^high^ Mals and enriched in invasive foci, and it was barely expressed in primary foci. On the other hand, analysis of an independent clinical cohort with a large sample size showed that SEMA3C expression correlated with tumor progression and the degree of metastasis, and higher levels of MVD were more common in PSCC samples with higher SEMA3C expression. Functional assays further supported the pro‐tumorigenic role of SEMA3C: knockdown of SEMA3C in PSCC cell lines significantly suppressed tumor sphere formation, invasion, and metastasis, while overexpression enhanced malignant phenotypes including migration and epithelial‐mesenchymal transition (EMT). Mechanistically, SEMA3C appears to serve as an important mediator of the interactions of SEMA3C^high^ Mals with other cells, particularly tip‐like capillary ECs. During tumor vascularization, tip ECs guide the growing sprout, and ECs subsequently differentiate into proliferative stalk cells, which are the main components of new vessels [68]. In this process, pericytes attach to ECs and support vasculature growth [69]. SEMA3C is capable of promoting the progression of a variety of tumors, including ovarian, lung, gastric, breast and prostate tumors [70, 71, 72, 73, 74]. Furthermore, our independent cohort data suggest that SEMA3C expression may hold prognostic relevance, as patients with elevated SEMA3C levels tended to exhibit poorer overall survival. This observation not only reinforces its functional role in tumor progression but also highlights the potential of SEMA3C as a biomarker to stratify patients with aggressive PSCC subtypes. While SEMA3C shows tumor‐associated specificity, its known physiological roles in neural and vascular development raise concerns about potential off‐tumor effects, which warrant further investigation in future translational studies [75]. Approaches such as tumor‐targeted delivery systems or context‐specific modulation of downstream pathways may help mitigate these risks [76].

Therefore, our study established a SEMA3C‐mediated malignant transformation model in PSCC. When PSCC is dominated by primary foci, SEMA3C is barely expressed, the MVD is low, and the tumor is in a relatively early stage. When SEMA3C is highly expressed in tumor cells, it can promote the interaction between tumor cells and ECs and promote the transformation of ECs from tip cells to stalk cells, leading to angiogenesis and ultimately promoting tumor progression and metastasis (Figure 7b). In summary, our study implies that SEMA3C may be an effective prognostic biomarker as well as a therapeutic target for PSCC.

## Author Contributions

X.C. had full access to all the data in the study and takes responsibility for the integrity of the data and the accuracy of the data analysis. X.C., M.Y., X.L., and X.H. contributed to the study concept and design. X.H. and W.S. contributed to the acquisition of data. X.H., W.S., and X.L. contributed to the analysis and interpretation of data. X.H., W.S., M.Y., X.C., and X.L. contributed to the drafting of the manuscript. L.D., X.Z., J.S., L.L., and T.L. contributed to the critical revision of the manuscript for important intellectual content. W.S. contributed to the statistical analysis. M.Y., X.H., and X.L. contributed to obtaining funding. X.H., M.Y., X.C., and X.L. contributed to the administrative, technical, or material support. X.C., M.Y., X.L., and X.H. contributed to the supervision. No other contributions were reported.

## Funding

This study was supported by National Key R&D Programmes (NKPs) of China (Grant No. 2022YFC3601800), Chongqing Wanzhou District PhD Direct‐Access Research Project (Grant No. wzstc‐20240006), Chongqing Medical Scientific Research Project (Joint Project of Chongqing Health Commission and Science and Technology Bureau; Grant No. 2025QNKX004), The Natural Science Foundation of Hunan Province of China (Grant No. 2023JJ30883), New Chongqing Youth Innovation Talent Project (Grant No. CSTB2025YITP‐QCRCX0005), The Natural Science Foundation of Hunan Province of China (Grant No. 2024JJ6703), Hunan Youth Talent Program for Distinguished Scholars (Grant No. 2024RC3045). The sponsors did not have any role in the study design, data collection, analysis, or interpretation, writing of the report, or the decision to submit the paper for publication.

## Conflicts of Interest

The authors declare no conflicts of interest.

## Supporting information




**Supporting File 1**: advs72682‐sup‐0001‐Figures.docx.

**Supporting File 2**: advs72682‐sup‐0002‐Table1.xlsx.

**Supporting File 3**: advs72682‐sup‐0003‐Table2.xlsx.

**Supporting File 4**: advs72682‐sup‐0004‐Table3.xlsx.

**Supporting File 5**: advs72682‐sup‐0005‐Table4.xlsx.

**Supporting File 6**: advs72682‐sup‐0006‐Table5.xlsx.

**Supporting File 7**: advs72682‐sup‐0007‐Table6.xlsx.

**Supporting File 8**: advs72682‐sup‐0008‐Table7.xlsx.

## Data Availability

The data supporting the conclusions drawn in this study are openly available in the Genome Sequence Archive (GSA) at https://ngdc.cncb.ac.cn/bioproject/, reference number PRJCA037152. Processed datasets is deposited in the OMIX data repository of the Chinese Academy of Sciences (https://ngdc.cncb.ac.cn/omix). The dataset is publicly available under accession number OMIX012250.

## References

[1] A. Thomas , A. Necchi , A. Muneer , et al., “Penile Cancer,” Nature Reviews Disease Primers 7, no. 1 (2021): 11. 10.1038/s41572-021-00246-5 33574340

[2] P. E. Clark , P. E. Spiess , N. Agarwal , et al., “Penile Cancer: Clinical Practice Guidelines in Oncology,” Journal of the National Comprehensive Cancer Network 11, no. 5 (2013): 594–615. 23667209 10.6004/jnccn.2013.0075 PMC4042432

[3] G. Agarwal , S. Gupta , and P. E. Spiess , “Novel Targeted Therapies for the Treatment of Penile Cancer,” Expert Opinion on Drug Discovery 9, no. 8 (2014): 959–968. 24896220 10.1517/17460441.2014.925875

[4] L. C. Pagliaro , D. L. Williams , D. Daliani , et al., “Neoadjuvant Paclitaxel, Ifosfamide, and Cisplatin Chemotherapy for Metastatic Penile Cancer: a Phase II Study,” Journal of Clinical Oncology 28, no. 24 (2010): 3851–3857. 20625118 10.1200/JCO.2010.29.5477 PMC2940402

[5] B. McGregor and G. Sonpavde , “Immunotherapy for Advanced Penile Cancer — Rationale and Potential,” Nature Reviews Urology 15, no. 12 (2018): 721–723. 10.1038/s41585-018-0083-0 30166593

[6] V. B. Joshi , P. E. Spiess , A. Necchi , C. A. Pettaway , and J. Chahoud , “Immune‐Based Therapies in Penile Cancer,” Nature Reviews Urology 19, no. 8 (2022): 457–474. 35851333 10.1038/s41585-022-00617-x

[7] T. F. Gajewski , H. Schreiber , and Y. X. Fu , “Innate and Adaptive Immune Cells in the Tumor Microenvironment,” Nature Immunology 14, no. 10 (2013): 1014–1022. 24048123 10.1038/ni.2703 PMC4118725

[8] G. Schneider , M. Schmidt‐Supprian , R. Rad , and D. Saur , “Tissue‐Specific Tumorigenesis: Context Matters,” Nature Reviews Cancer 17, no. 4 (2017): 239–253. 28256574 10.1038/nrc.2017.5 PMC5823237

[9] S. Shang , M.‐Z. Wang , Z. Xing , N. He , and S. Li , “Lactate Regulators Contribute to Tumor Microenvironment and Predict Prognosis in Lung Adenocarcinoma,” Frontiers in Immunology 13 (2022): 1024925. 36505423 10.3389/fimmu.2022.1024925 PMC9732022

[10] D. F. Quail and J. A. Joyce , “Microenvironmental Regulation of Tumor Progression and Metastasis,” Nature Medicine 19, no. 11 (2013): 1423–1437. 10.1038/nm.3394 PMC3954707 24202395

[11] X. Mao , J. Xu , W. Wang , et al., “Crosstalk between Cancer‐Associated Fibroblasts and Immune Cells in the Tumor Microenvironment: New Findings and Future Perspectives,” Molecular Cancer 20, no. 1 (2021): 131. 34635121 10.1186/s12943-021-01428-1 PMC8504100

[12] D. Grün and A. van Oudenaarden , “Design and Analysis of Single‐Cell Sequencing Experiments,” Cell 163, no. 4 (2015): 799–810. 26544934 10.1016/j.cell.2015.10.039

[13] L. Elst , G. Philips , K. Vandermaesen , et al., “Single‐Cell Atlas of Penile Cancer Reveals TP53 Mutations as a Driver of an Aggressive Phenotype, Irrespective of Human Papillomavirus Status, and Provides Clues for Treatment Personalization,” European Urology 86, no. 2 (2024): 114–127. 38670879 10.1016/j.eururo.2024.03.038

[14] H. Song , T. Zhichao , X. Guixiang , et al., “Single‐Cell and Spatial Transcriptomic Profiling of Penile Squamous Cell Carcinoma Reveals Dynamics of Tumor Differentiation and Immune Microenvironment,” Advanced Science 12 (2025): 00216. 10.1002/advs.202500216 PMC12412502 40470730

[15] G. S. Gulati , S. S. Sikandar , D. J. Wesche , et al., “Single‐Cell Transcriptional Diversity Is a Hallmark of Developmental Potential,” Science 367, no. 6476 (2020): 405–411. 31974247 10.1126/science.aax0249 PMC7694873

[16] B. Li , E. Severson , J.‐C. Pignon , et al., “Comprehensive Analyses of Tumor Immunity: Implications for Cancer Immunotherapy,” Genome Biology 17, no. 1 (2016): 174. 27549193 10.1186/s13059-016-1028-7 PMC4993001

[17] D. Szklarczyk , A. L. Gable , D. Lyon , et al., “STRING v11: Protein–protein Association Networks with Increased Coverage, Supporting Functional Discovery in Genome‐Wide Experimental Datasets,” Nucleic Acids Research 47, no. D1 (2019): D607–D613. 30476243 10.1093/nar/gky1131 PMC6323986

[18] I. Korsunsky , N. Millard , J. Fan , et al., “Fast, Sensitive and Accurate Integration of Single‐Cell Data with Harmony,” Nature Methods 16, no. 12 (2019): 1289–1296. 31740819 10.1038/s41592-019-0619-0 PMC6884693

[19] T. Huang , X. Cheng , J. Chahoud , et al., “Effective Combinatorial Immunotherapy for Penile Squamous Cell Carcinoma,” Nature Communications 11, no. 1 (2020): 2124. 10.1038/s41467-020-15980-9 PMC7195486 32358507

[20] I. Trias , A. Saco , L. Marimon , et al., “P53 in Penile Squamous Cell Carcinoma: a Pattern‐Based Immunohistochemical Framework with Molecular Correlation,” Cancers 15, no. 10 (2023): 2719. 37345055 10.3390/cancers15102719 PMC10216449

[21] J. J. Muñoz , S. A. Drigo , H. Kuasne , et al., “A Comprehensive Characterization of Cell Cultures and Xenografts Derived from a human Verrucous Penile Carcinoma,” Tumor Biology 37, no. 8 (2016): 11375–11384. 26960831 10.1007/s13277-016-4951-z

[22] X. Lei , Y. Lei , J.‐K. Li , et al., “Immune Cells within the Tumor Microenvironment: Biological Functions and Roles in Cancer Immunotherapy,” Cancer Letters 470 (2020): 126–133. 31730903 10.1016/j.canlet.2019.11.009

[23] S. Gordon , “Alternative Activation of Macrophages,” Nature Reviews Immunology 3, no. 1 (2003): 23–35. 10.1038/nri978 12511873

[24] S. Cheng , Z. Li , R. Gao , et al., “A Pan‐Cancer Single‐Cell Transcriptional Atlas of Tumor Infiltrating Myeloid Cells,” Cell 184, no. 3 (2021): 792–809.e23. 33545035 10.1016/j.cell.2021.01.010

[25] R. J. Johnston , L. Comps‐Agrar , J. Hackney , et al., “The Immunoreceptor TIGIT Regulates Antitumor and Antiviral CD8^+^ T Cell Effector Function,” Cancer Cell 26, no. 6 (2014): 923–937. 25465800 10.1016/j.ccell.2014.10.018

[26] X. Chen and E. Song , “Turning Foes to Friends: Targeting Cancer‐Associated Fibroblasts,” Nature Reviews Drug Discovery 18, no. 2 (2019): 99–115. 30470818 10.1038/s41573-018-0004-1

[27] D. Park , E. Sahai , and A. Rullan , “SnapShot: Cancer‐Associated Fibroblasts,” Cell 181, no. 2 (2020): 486–486.e1. 32302576 10.1016/j.cell.2020.03.013

[28] D. Öhlund , A. Handly‐Santana , G. Biffi , et al., “Distinct Populations of Inflammatory Fibroblasts and Myofibroblasts in Pancreatic Cancer,” Journal of Experimental Medicine 214, no. 3 (2017): 579–596. 28232471 10.1084/jem.20162024 PMC5339682

[29] A. Zirkel , M. Lederer , N. Stöhr , N. Pazaitis , and S. Hüttelmaier , “IGF2BP1 promotes Mesenchymal Cell Properties and Migration of Tumor‐Derived Cells by Enhancing the Expression of LEF1 and SNAI2 (SLUG),” Nucleic Acids Research 41, no. 13 (2013): 6618–6636. 23677615 10.1093/nar/gkt410 PMC3711427

[30] Z. Pan , T. Xu , L. Bao , et al., “CREB3L1 promotes Tumor Growth and Metastasis of Anaplastic Thyroid Carcinoma by Remodeling the Tumor Microenvironment,” Molecular Cancer 21, no. 1 (2022): 190. 36192735 10.1186/s12943-022-01658-x PMC9531463

[31] Z.‐L. Cai , B. Shen , Y. Yuan , et al., “The Effect of HMGA1 in LPS‐Induced Myocardial Inflammation,” International Journal of Biological Sciences 16, no. 11 (2020): 1798–1810. 32398950 10.7150/ijbs.39947 PMC7211173

[32] D. Qi , M. Lu , P. Xu , et al., “Transcription Factor ETV4 Promotes the Development of Hepatocellular Carcinoma by Driving Hepatic TNF‐α Signaling,” Cancer Communications 43, no. 12 (2023): 1354–1372. 37670477 10.1002/cac2.12482 PMC10693303

[33] S. Jin , C. F. Guerrero‐Juarez , L. Zhang , et al., “Inference and Analysis of Cell‐Cell Communication Using CellChat,” Nature Communications 12, no. 1 (2021): 1088. 10.1038/s41467-021-21246-9 PMC7889871 33597522

[34] X. Liu , L. Meng , X. Li , et al., “Correction: Regulation of FN1 Degradation by p62/SQSTM1‐Dependent Autophagy–lysosomal Pathway in HNSCC,” International Journal of Oral Science 13, no. 1 (2021): 34. 34728607 10.1038/s41368-021-00139-z PMC8564542

[35] P. Karpinski , I. Rosales , L. Laczmanski , A. Kowalik , S. Wenson , and M. P. Hoang , “Expression of Genes Associated with Epithelial‐Mesenchymal Transition in Merkel Cell Polyomavirus–Negative Merkel Cell Carcinoma,” Laboratory Investigation 103, no. 8 (2023): 100177. 37207705 10.1016/j.labinv.2023.100177

[36] H. Hamidi and J. Ivaska , “Every Step of the Way: Integrins in Cancer Progression and Metastasis,” Nature Reviews Cancer 18, no. 9 (2018): 533–548. 30002479 10.1038/s41568-018-0038-z PMC6629548

[37] J. W. Slaton , N. Morgenstern , D. A. Levy , et al., “Tumor Stage, Vascular Invasion and the Percentage of Poorly Differentiated Cancer: Independent Prognosticators for Inguinal Lymph Node Metastasis in Penile Squamous Cancer,” Journal of Urology 165, no. 4 (2001): 1138–1142. 11257655

[38] X. Pan , X. Li , L. Dong , et al., “Tumour Vasculature at Single‐Cell Resolution,” Nature 632, no. 8024 (2024): 429–436. 38987599 10.1038/s41586-024-07698-1

[39] L.‐A. Teuwen , L. P. M. H. De Rooij , A. Cuypers , et al., “Tumor Vessel Co‐Option Probed by Single‐Cell Analysis,” Cell Reports 35, no. 11 (2021): 109253. 34133923 10.1016/j.celrep.2021.109253

[40] M. Chekenya , M. Hjelstuen , P. Enger , et al., “NG_2_ proteoglycan Promotes Angiogenesis‐Dependent Tumor Growth in the central Nervous System by Sequestering Angiostatin,” The FASEB Journal 16, no. 6 (2002): 586–588. 11919162 10.1096/fj.01-0632fje

[41] Q. Zhang , Y. He , N. Luo , et al., “Landscape and Dynamics of Single Immune Cells in Hepatocellular Carcinoma,” Cell 179, no. 4 (2019): 829–845.e20. 31675496 10.1016/j.cell.2019.10.003

[42] L. Zhang , Z. Li , K. M. Skrzypczynska , et al., “Single‐Cell Analyses Inform Mechanisms of Myeloid‐Targeted Therapies in Colon Cancer,” Cell 181, no. 2 (2020): 442–459.e29. 32302573 10.1016/j.cell.2020.03.048

[43] L. Hlatky , P. Hahnfeldt , and J. Folkman , “Clinical Application of Antiangiogenic Therapy: Microvessel Density, What It Does and Doesn't Tell Us,” Journal of the National Cancer Institute 94, no. 12 (2002): 883–893. 12072542 10.1093/jnci/94.12.883

[44] B. Dura , J.‐Y. Choi , K. Zhang , et al., “scFTD‐seq: Freeze‐Thaw Lysis Based, Portable Approach toward Highly Distributed Single‐Cell 3′ mRNA Profiling,” Nucleic Acids Research 47, no. 3 (2019): 16. 10.1093/nar/gky1173 PMC6379653 30462277

[45] A. Kechin , U. Boyarskikh , A. Kel , and M. Filipenko , “cutPrimers: a New Tool for Accurate Cutting of Primers from Reads of Targeted Next Generation Sequencing,” Journal of Computational Biology 24, no. 11 (2017): 1138–1143. 28715235 10.1089/cmb.2017.0096

[46] A. Dobin , C. A. Davis , F. Schlesinger , et al., “STAR: Ultrafast Universal RNA‐seq Aligner,” Bioinformatics 29, no. 1 (2013): 15–21. 23104886 10.1093/bioinformatics/bts635 PMC3530905

[47] Y. Liao , G. K. Smyth , and W. Shi , “featureCounts: an Efficient General Purpose Program for Assigning Sequence Reads to Genomic Features,” Bioinformatics 30, no. 7 (2014): 923–930. 24227677 10.1093/bioinformatics/btt656

[48] S. L. Wolock , R. Lopez , and A. M. Klein , “Scrublet: Computational Identification of Cell Doublets in Single‐Cell Transcriptomic Data,” Cell Systems 8, no. 4 (2019): 281–291.e9. 30954476 10.1016/j.cels.2018.11.005 PMC6625319

[49] G. Yu , L.‐G. Wang , Y. Han , and Q.‐Y. He , “clusterProfiler: an R Package for Comparing Biological Themes among Gene Clusters,” OMICS: A Journal of Integrative Biology 16, no. 5 (2012): 284–287. 22455463 10.1089/omi.2011.0118 PMC3339379

[50] Y. Zhou , B. Zhou , L. Pache , et al., “Metascape Provides a Biologist‐Oriented Resource for the Analysis of Systems‐Level Datasets,” Nature Communications 10, no. 1 (2019): 1523. 10.1038/s41467-019-09234-6 PMC6447622 30944313

[51] C. L. Plaisier , M. Pan , and N. S. Baliga , “A miRNA‐Regulatory Network Explains How Dysregulated miRNAs Perturb Oncogenic Processes across Diverse Cancers,” Genome Research 22, no. 11 (2012): 2302–2314. 22745231 10.1101/gr.133991.111 PMC3483559

[52] L. Zheng , S. Qin , W. Si , et al., “Pan‐Cancer Single‐Cell Landscape of Tumor‐Infiltrating T Cells,” Science 374, no. 6574 (2021): abe6474. 34914499 10.1126/science.abe6474

[53] S. Hanzelmann , R. Castelo , and J. Guinney , “GSVA: Gene Set Variation Analysis for Microarray and RNA‐seq Data,” BMC Bioinformatics 14 (2013): 7. 23323831 10.1186/1471-2105-14-7 PMC3618321

[54] X. Qiu , A. Hill , J. Packer , D. Lin , Y.‐A. Ma , and C. Trapnell , “Single‐Cell mRNA Quantification and Differential Analysis with Census,” Nature Methods 14, no. 3 (2017): 309–315. 28114287 10.1038/nmeth.4150 PMC5330805

[55] C. Trapnell , D. Cacchiarelli , J. Grimsby , et al., “The Dynamics and Regulators of Cell Fate Decisions Are Revealed by Pseudotemporal Ordering of Single Cells,” Nature Biotechnology 32, no. 4 (2014): 381–386. 10.1038/nbt.2859 PMC4122333 24658644

[56] Q.‐H. Zhou , C.‐Z. Deng , Z.‐S. Li , et al., “Molecular Characterization and Integrative Genomic Analysis of a Panel of Newly Established Penile Cancer Cell Lines,” Cell Death & Disease 9, no. 6 (2018): 684. 29880898 10.1038/s41419-018-0736-1 PMC5992159

[57] X. Hu , M. Chen , Y. Li , Y. Wang , S. Wen , and F. Jun , “Overexpression of ID1 Promotes Tumor Progression in Penile Squamous Cell Carcinoma,” Oncology Reports 41, no. 2 (2019): 1091–1100. 30535485 10.3892/or.2018.6912

[58] X. Hu , M. Chen , W. Liu , Y. Li , and J. Fu , “Preoperative Plasma IGFBP2 Is Associated with Nodal Metastasis in Patients with Penile Squamous Cell Carcinoma,” Urologic Oncology: Seminars and Original Investigations 37, no. 7 (2019): 452–461. 31053522 10.1016/j.urolonc.2019.04.013

[59] S. J. Mills , A. J. Cowin , and P. Kaur , “Pericytes, Mesenchymal Stem Cells and the Wound Healing Process,” Cells 2, no. 3 (2013): 621–634. 24709801 10.3390/cells2030621 PMC3972668

[60] Y. Cao , “Angiogenesis and Vascular Functions in Modulation of Obesity, Adipose Metabolism, and Insulin Sensitivity,” Cell Metabolism 18, no. 4 (2013): 478–489. 24035587 10.1016/j.cmet.2013.08.008

[61] Y.‐T. Chen , F.‐C. Chang , C.‐F. Wu , et al., “Platelet‐Derived Growth Factor Receptor Signaling Activates Pericyte–myofibroblast Transition in Obstructive and Post‐Ischemic Kidney Fibrosis,” Kidney International 80, no. 11 (2011): 1170–1181. 21716259 10.1038/ki.2011.208

[62] J. Winkler , A. Abisoye‐Ogunniyan , K. J. Metcalf , and Z. Werb , “Concepts of Extracellular Matrix Remodelling in Tumour Progression and Metastasis,” Nature Communications 11, no. 1 (2020): 5120. 10.1038/s41467-020-18794-x PMC7547708 33037194

[63] K. M. Ilieva , A. Cheung , S. Mele , et al., “Chondroitin Sulfate Proteoglycan 4 and Its Potential as an Antibody Immunotherapy Target across Different Tumor Types,” Frontiers in Immunology 8 (2017): 1911. 29375561 10.3389/fimmu.2017.01911 PMC5767725

[64] P. A. Nicolosi , A. Dallatomasina , and R. Perris , “Theranostic Impact of NG2/CSPG4 Proteoglycan in Cancer,” Theranostics 5, no. 5 (2015): 530–544. 25767619 10.7150/thno.10824 PMC4350014

[65] R. Warta , C. Herold‐Mende , J. Chaisaingmongkol , et al., “Reduced Promoter Methylation and Increased Expression of CSPG4 Negatively Influences Survival of HNSCC Patients,” International Journal of Cancer 135, no. 11 (2014): 2727–2734. 24740185 10.1002/ijc.28906

[66] M. A. Price , L. E. Colvin Wanshura , J. Yang , et al., “CSPG4, a Potential Therapeutic Target, Facilitates Malignant Progression of Melanoma,” Pigment Cell & Melanoma Research 24, no. 6 (2011): 1148–1157. 22004131 10.1111/j.1755-148X.2011.00929.x PMC3426219

[67] L.‐L. Lu , “Neuron‐Glial Antigen 2 Overexpression in Hepatocellular Carcinoma Predicts Poor Prognosis,” World Journal of Gastroenterology 21, no. 21 (2015): 6649–59. 26074703 10.3748/wjg.v21.i21.6649 PMC4458775

[68] H. W. Lee , J. H. Shin , and M. Simons , “Flow Goes Forward and Cells Step Backward: Endothelial Migration,” Experimental & Molecular Medicine 54, no. 6 (2022): 711–719. 35701563 10.1038/s12276-022-00785-1 PMC9256678

[69] A. Y. Liu and G. Ouyang , “Tumor Angiogenesis: a New Source of Pericytes,” Current Biology 23, no. 13 (2013): R565–R568. 23845244 10.1016/j.cub.2013.05.023

[70] T. Yamada , R. Endo , M. Gotoh , and S. Hirohashi , “Identification of Semaphorin E as a Non‐MDR Drug Resistance Gene of human Cancers,” Proceedings of the National Academy of Sciences 94, no. 26 (1997): 14713–14718. 10.1073/pnas.94.26.14713 PMC25101 9405678

[71] M. Martín‐Satué and J. Blanco , “Identification of Semaphorin E Gene Expression in Metastatic human Lung Adenocarcinoma Cells by mRNA Differential Display,” Journal of Surgical Oncology 72, no. 1 (1999): 18–23. 10477871 10.1002/(sici)1096-9098(199909)72:1<18::aid-jso5>3.0.co;2-p

[72] H. Miyato , N. H. Tsuno , and J. Kitayama , “Semaphorin 3C Is Involved in the Progression of Gastric Cancer,” Cancer Science 103, no. 11 (2012): 1961–1966. 22924992 10.1111/cas.12003 PMC7659286

[73] C. Esselens , J. Malapeira , N. Colomé , et al., “The Cleavage of Semaphorin 3C Induced by ADAMTS1 Promotes Cell Migration,” Journal of Biological Chemistry 285, no. 4 (2010): 2463–2473. 19915008 10.1074/jbc.M109.055129 PMC2807303

[74] J. G. Herman and G. G. Meadows , “Increased Class 3 Semaphorin Expression Modulates the Invasive and Adhesive Properties of Prostate Cancer Cells,” International Journal of Oncology 30, no. 5 (2007): 1231–1238. 17390026

[75] C. Gu and E. Giraudo , “The Role of Semaphorins and Their Receptors in Vascular Development and Cancer,” Experimental Cell Research 319, no. 9 (2013): 1306–1316. 23422037 10.1016/j.yexcr.2013.02.003 PMC3648602

[76] M. Barok , H. Joensuu , and J. Isola , “Trastuzumab Emtansine: Mechanisms of Action and Drug Resistance,” Breast Cancer Research 16, no. 2 (2014): 209. 24887180 10.1186/bcr3621 PMC4058749

