# Supplementary material for: Decoding the Cellular Heterogeneity and Malignant Progression of Human Penile Squamous Cell Carcinoma by Single‐Cell RNA Sequencing
Source: Adv Sci (Weinh). 2026 Feb 4;13(14):e03894. doi: 10.1002/advs.202503894 (PMC12970255; doi:10.1002/advs.202503894)
Supplement: Supplementary file 1 — Supporting File 1: advs72682‐sup‐0001‐Figures.docx. [file ADVS-13-e03894-s005.docx]

**Decoding the cellular heterogeneity and malignant progression of human penile squamous cell carcinoma by single-cell RNA sequencing**

Xiheng Hu^1,2,3,4#*^**,** Wensheng Shi^1,6#^, Liang Dong^2,3,4^, Lingjuan Huang^2,3,4^, Xiyuan Zhang^2,3,4^, Yiting Feng^2,3,4^, Jie Sun^9,10,11^, Lanlan Liu^9,10,11^, Teng Liu^9,10,11^, Jun Fu^9,10,11^, Bowen Zhong^1^, Qihao Leng^1^, Xiaohua Wu^1^, Minfeng Chen^1^, Lingfang Li^5^, Yuan Li^6^, Xin Jin^6^, Long Wang^7^, Jian Cao^8^**,** Xin Li^9,10,11*^, Mingzhu Yin^9,10,11*^, Xiang Chen^2,3,4*^

1 Department of Urology, Xiangya Hospital, Central South University, Changsha, Hunan, China

2 National Engineering Research Center of Personalized Diagnostic and Therapeutic Technology, Central South University, Changsha, Hunan, China

3 Furong Laboratory, Changsha, Hunan, China

4 Department of Dermatology, Hunan Engineering Research Center of Skin Health

and Disease, Hunan Key Laboratory of Skin Cancer and Psoriasis, Xiangya Hospital, Central South University, Changsha, Hunan, China

5 Department of Cardiovascular Medicine, Xiangya Hospital, Central South University, Changsha, China

6 Department of Urology, the Second Xiangya Hospital, Central South University, China

7 Department of Urology, The Third Xiangya Hospital of Central South University, Changsha, Hunan 410013, China

8 Department of Urology, The Affiliated Cancer Hospital of Xiangya School of Medicine, Central South University/Hunan Cancer Hospital, Changsha, China

9 [Clinical Research Center](javascript:;) (CRC), Medical Pathology Center (MPC), Cancer Early Detection and Treatment Center (CEDTC)，Chongqing University Three Gorges Hospital, Chongqing University, Chongqing, China

10 Translational Medicine Research Center (TMRC), School of Medicine Chongqing University, Chongqing, China

11 Chongqing University Three Gorges Hospital & Academy for Advanced interdisciplinary Technology, CQU - Ferenc Krausz Nobel Laureate Scientific Workstation, Chongqing, China

^#^ These authors contributed equally to this work.

**^*^Corresponding authors.**

Xiang Chen, ORCID number: 0000-0001-8187-636X; Tel: +86 731-88879282; Fax:

+86 731-88710591; mail: Xiang Chen, [chenxiangck@126.com](mailto:chenxiangck@126.com); or Mingzhu Yin, yinmingzhu2008@126.com； or Xin Li，lixin920126@163.com； or Xiheng Hu，403269833@qq.com


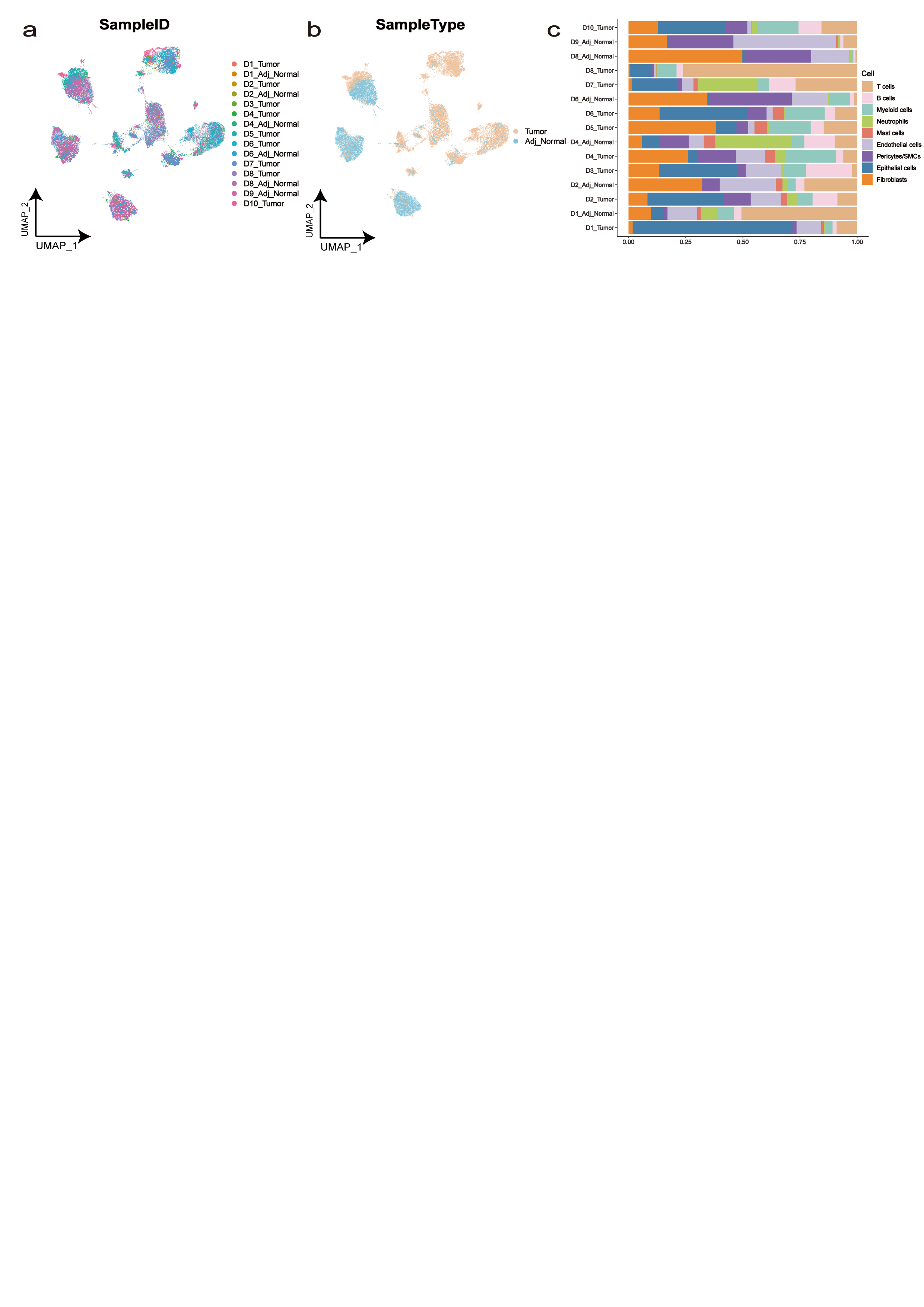


**Fig. S1 Visualization of single-cell transcriptomes. a,b** UMAP embedding of 66,421 high-quality cells, colored by SampleID (a) or SampleType (b). **c** The proportion of cells from each cluster type in each sample.

**
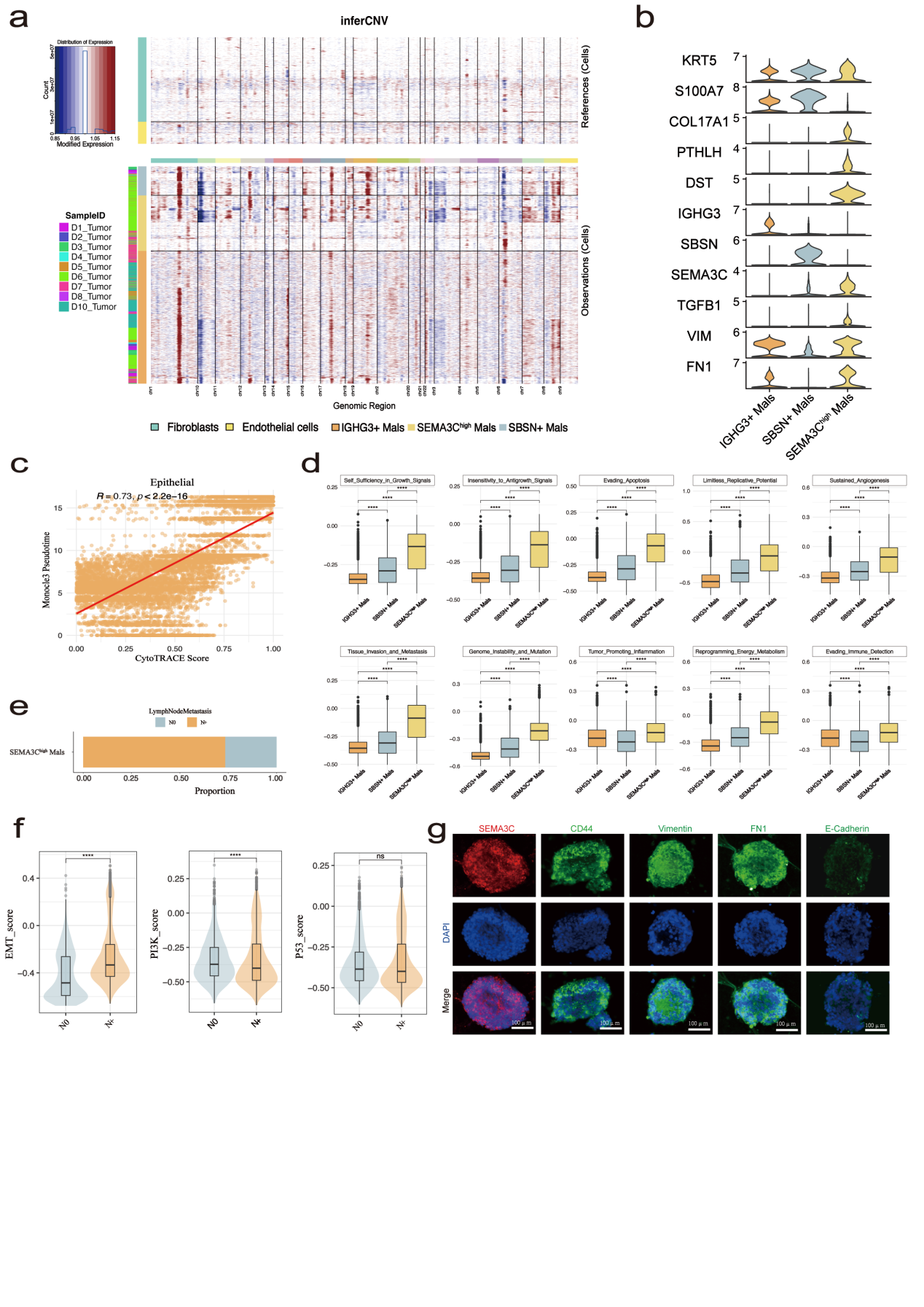
**

**Fig. S2 Molecular features of malignant epithelial cells in PSCC. a** Prediction of malignant cells by inferCNV analysis. Normal fibroblasts and endothelial cells were used as the reference population. **b** Violin plot showing the expression level of representative epithelial and malignant markers. Expression values were computed using Seurat's NormalizeData() function with log1p transformation. **c** Correlation analysis between pseudotime values inferred by Monocle3 and differentiation scores generated by CytoTRACE. **d** The GSVA scores of “hallmarks of cancer” between normal epithelial cells and malignant cells, Wilcoxon signed-rank test, ^****^*p* < 0.0001. **e** Distribution of SEMA3C^high^ Mals across PSCC patients with different lymph node metastasis status. **f** Comparison of EMT score, PI3K score and P53 score between different lymph node metastasis status, Wilcoxon signed-rank test, *****p* < 0.0001. **g** Immunofluorescence staining of SEMA3C, VIM (Vimentin), CD44, FN1 (Fibronectin 1), and E-Cadherin in tumour spheres. Scale bar, 100 μm.


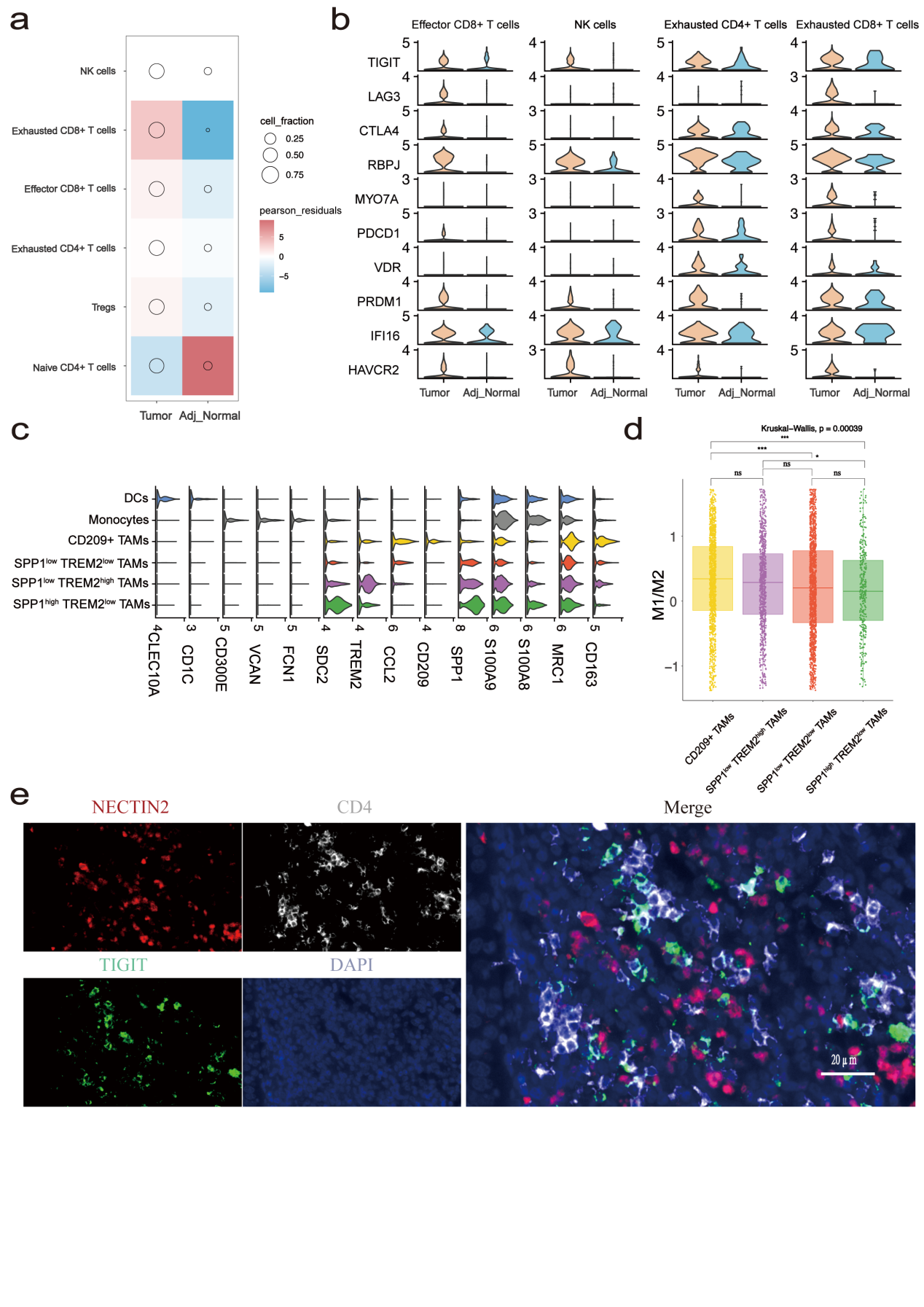


**Fig. S3 Marker genes of immune cells. a** Pearson residuals and cell fraction of each cell cluster in different sample types. **b** Expression of exhaustion-related markers in different cells across various samples. Expression values were computed using Seurat's NormalizeData() function with log1p transformation. **c** Violin plot of representative genes in myeloid cells. Expression values were computed using Seurat's NormalizeData() function with log1p transformation. **d** Comparison of M1/M2 score among four kinds of TAMs, ranking by the median value. **e** Immunofluorescence staining of NECTIN2 (red), TIGIT (green), and CD4 (white) in tumour sections, showing spatial co-localization between NECTIN2^+^ TAMs and TIGIT^+^ CD4^+^ T cells. Nuclei were counterstained with DAPI (blue). Scale bar, 20 μm.

**
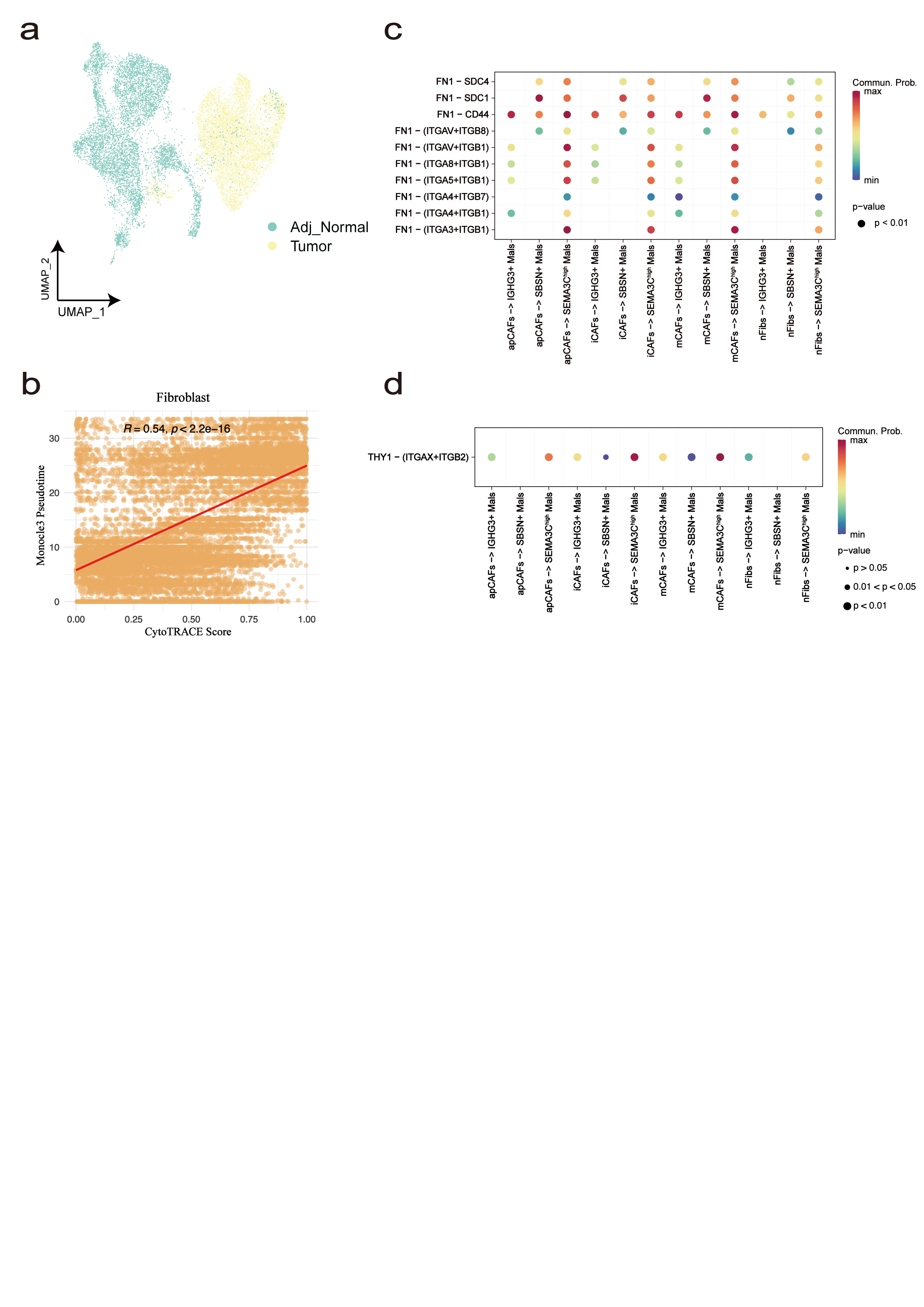
**

**Fig. S4** **Cell-cell interactions between CAFs and other cells. a** The UMAP plot shows the distribution of fibroblasts across different samples. **b** Correlation analysis between pseudotime values inferred by Monocle3 and differentiation scores generated by CytoTRACE. **c** Ligand-receptor interactions of FN1 signalling pathway. **d** Ligand-receptor interactions of THY1 signalling pathway. The size of circle reflects *p* values and the color gradient indicate the communication probability of the interaction.


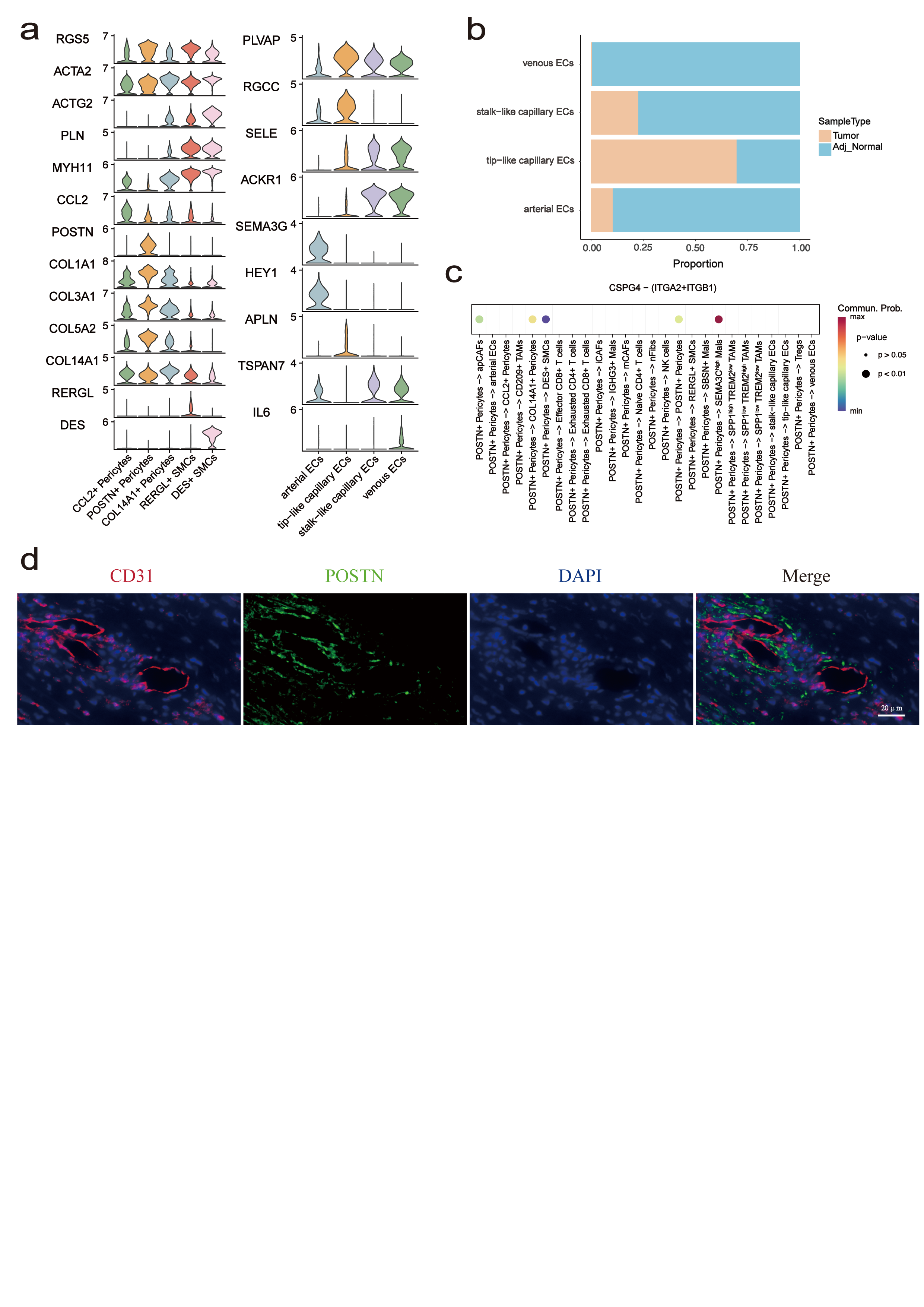


**Fig. S5 Functional analysis of POSTN^+^ pericytes. a** Violin plot of representative genes in pericytes/SMCs and ECs. Expression values were computed using Seurat's NormalizeData() function with log1p transformation. **b** The proportion of cells from each sample type in each cluster. **c** Ligand-receptor interactions of CSPG4 signalling pathway in POSTN^+^ pericytes, the size of circle reflects *p* values and the color gradient indicate the communication probability of the interaction. **d** Immunofluorescence staining of CD31 (red), POSTN (green), and DAPI (blue) in tumour tissue. Scale bar, 20 μm.


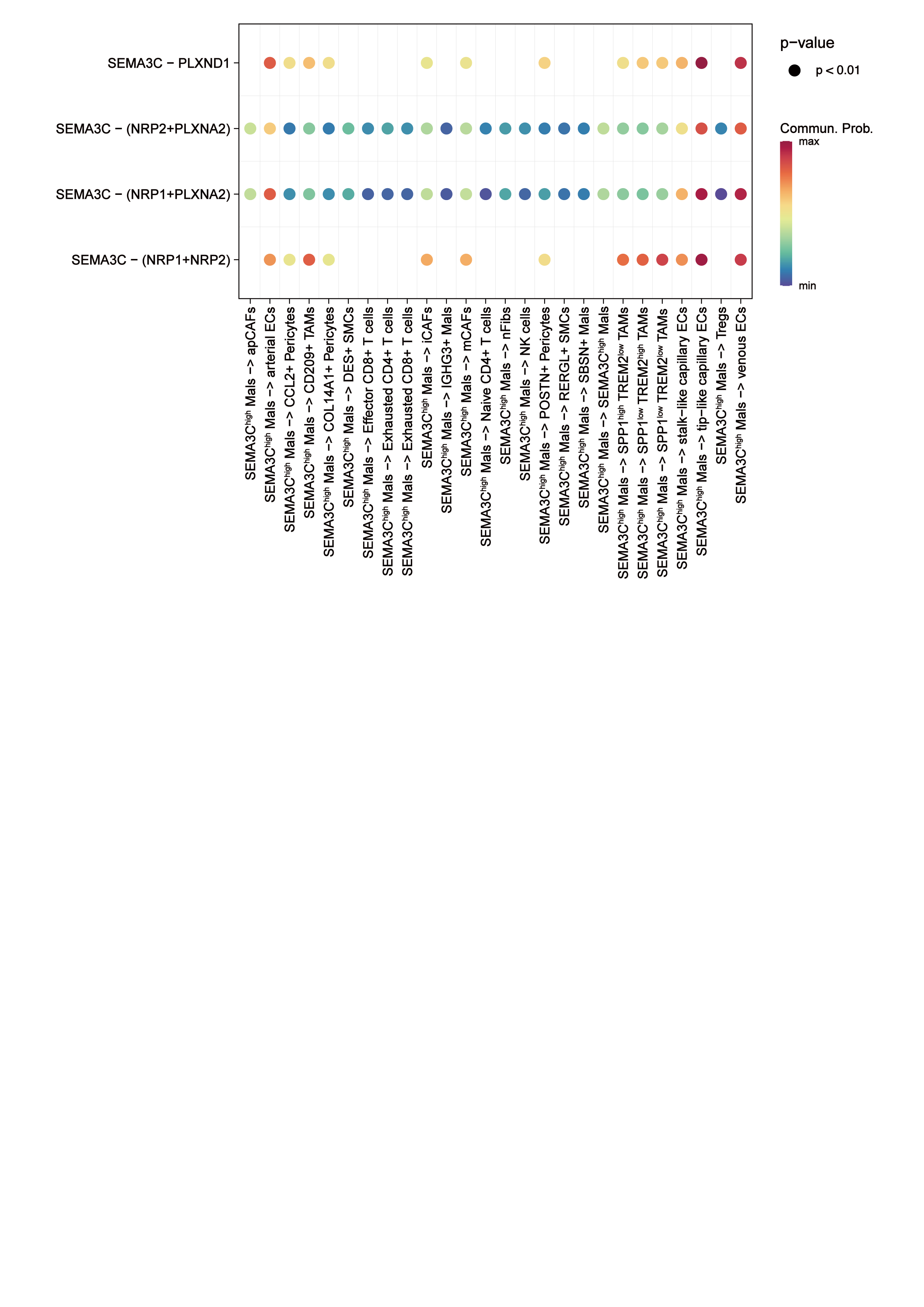


**Fig. S6** **Ligand-receptor interactions of SEMA3 signalling pathway in PSCC, the size of circle reflects *p* values and the color gradient indicate the communication probability of the interaction.**

**
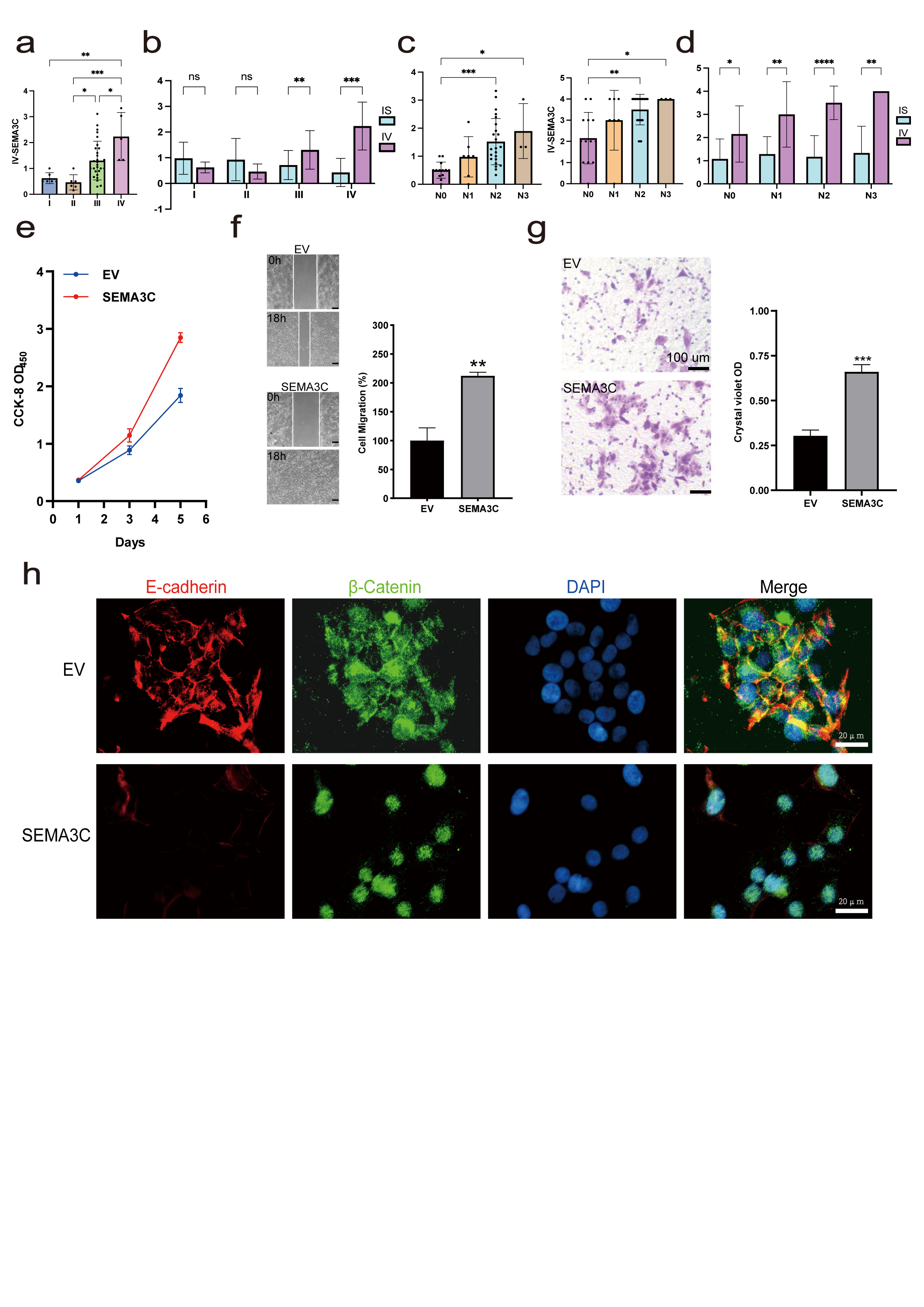
**

**Fig. S7** **Functional Characterization and Clinical Implication of SEMA3C in PSCC. a** The expression score of SEMA3C in IV among different TNM staging, n=47. **b** The expression score of SEMA3C in IS or IV among different TNM staging, n=47. **c** The picture in the left show the expression score of SEMA3C in different N staging, the picture in the right show the the expression score of SEMA3C in IV among different N staging, n=47. **d** The expression score of SEMA3C in IS or IV among different N staging, n=47. Wilcoxon signed-rank test, ^*^*p* < 0.05, ^***^*p* < 0.001, ^****^*p* < 0.0001. **e** CCK-8 assay showing enhanced proliferation of 149Rca cells overexpressing SEMA3C compared to empty vector (EV) control over a 6-day period. **f** Representative images and quantification of wound healing assays at 0 h and 18 h showing increased migration in SEMA3C-overexpressing cells. **g** Transwell invasion assay showing a significant increase in invasive capacity upon SEMA3C overexpression; right panel quantifies crystal violet staining (OD values). Scale bar, 100 μm. **h** Immunofluorescence staining of E-cadherin (red), β-Catenin (green), and DAPI (blue) showing loss of epithelial markers and cytoplasmic redistribution of β-Catenin in SEMA3C-overexpressing cells. Scale bar, 20 μm.


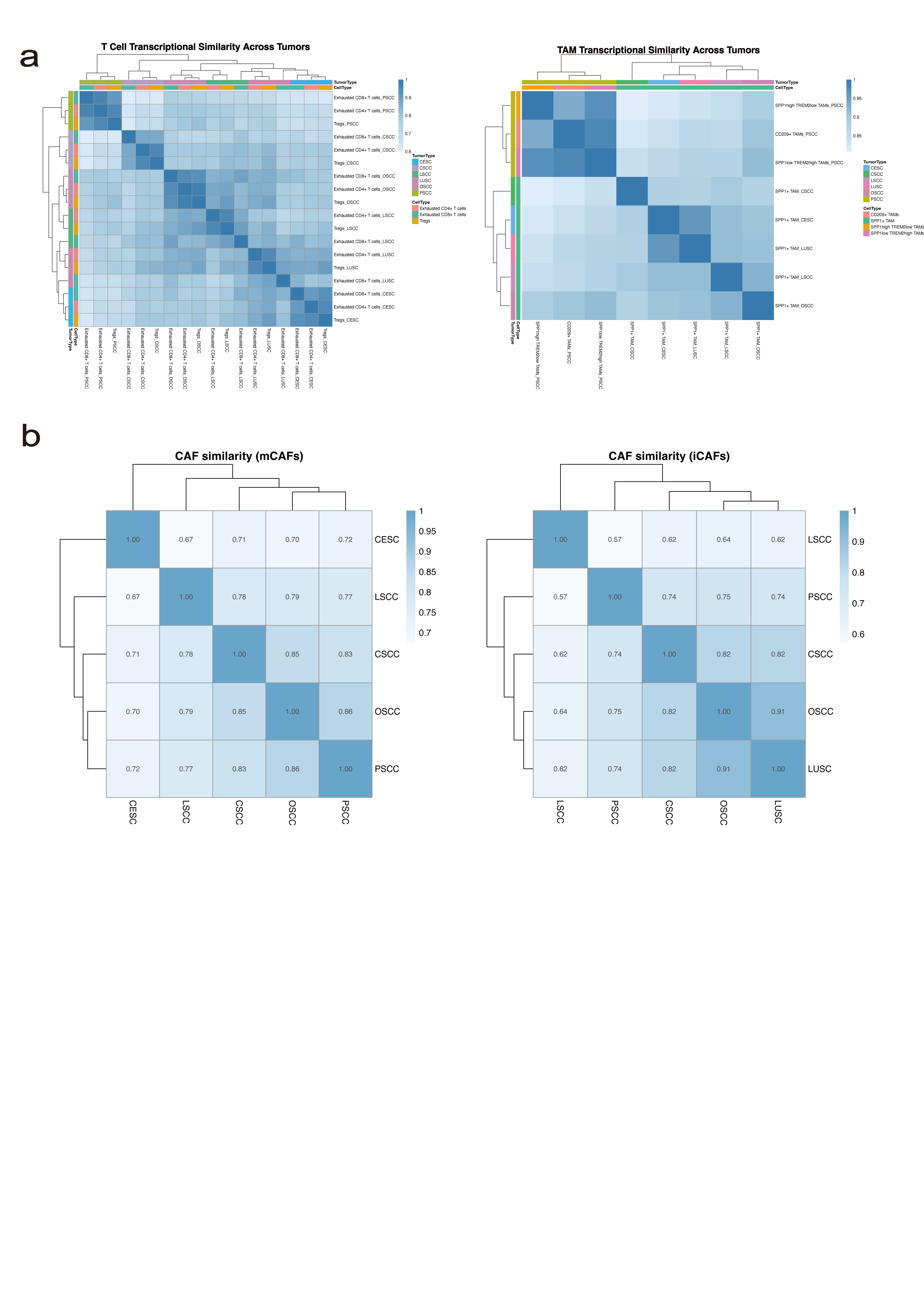


**Fig. S8** **Cross-cancer transcriptomic similarity of key TME cell subsets among squamous cell carcinomas. a** Heatmaps showing transcriptomic similarity of T cells (left) and TAMs (right) across PSCC and other squamous cell carcinomas, including CESC, CSCC, OSCC, LSCC, and LUSC. Similarity scores were calculated based on gene expression profiles and visualized with hierarchical clustering. **b** Similarity matrices of myofibroblastic CAFs (mCAFs, left) and inflammatory CAFs (iCAFs, right) across PSCC and four additional SCC types.
